# Ultrasound and Microwave Treatments to Produce Flexible Thermoplastic Starch–Brewers’ Spent Grain Composite Films

**DOI:** 10.3390/polym18080967

**Published:** 2026-04-16

**Authors:** Antonietta Baiano, Antonella Di Palma, Anna Fiore

**Affiliations:** Dipartimento di Scienze Agrarie, Alimenti, Risorse Naturali e Ingegneria (DAFNE), University of Foggia, Via Napoli 25, 71122 Foggia, Italy; antonella.dipalma@unifg.it (A.D.P.); anna.fiore@unifg.it (A.F.)

**Keywords:** brewers spent grain (BSG), by-product, circular economy, film, microwave, sustainable packaging, ultrasound

## Abstract

This research aimed to evaluate the effects of formulation and process conditions on the physical and structural properties of starch–brewers’ spent grain films. Three factors were considered: BSG amounts (0, 1, 3, 5%), a possible ultrasonication pre-treatment, and different microwave gelatinization treatments (450 W for 80 and 90 s; 900 W for 45 and 50 s). An increase in BSG is responsible for increases in moisture (10.72 → 23.40%), water absorption (67.65 → 95.73%), density (0.90 → 1.27 g/cm^3^), browning index (5.86 → 85.88), UV blocking capacity (82.42% → 99.96% for UV_A; 61.28% → 99.86% for UV_B), and degradability in the first 7 days (58.72 → 66.57%), but dramatically decreases the Young’s modulus and tensile strength (fallen to 2.90 N/mm^2^ and 0.21 N/mm^2^, at 5% BSG). Sonication contributes to increased browning index (36.17 → 37.24), UV blocking capacity, solubility (49.35 → 51.49%) and Young’s modulus (4.40 → 4.77 N/mm^2^). The most severe microwave treatment (900 W, 50 s) minimizes moisture (15.83%) and water absorption (80.89%) and maximizes density (1.21 g/cm^3^), browning index (37.52), and Young’s modulus (5.37 N/mm^2^). SEM micrographs allow us to observe that the film surface appears rough, and the structure becomes increasingly porous as BSG % increases. The regression analysis indicates that the quadratic model effectively describes the relationships between the three factors and each of the most important properties of the films; it is suitable for predicting film behavior and optimizing their characteristics depending on the desired use.

## 1. Introduction

A critical element in sustainable food consumption is packaging. It must be able to both ensure food safety and quality and address the need for reductions in food loss and waste. Beyond these basic requirements, a new challenge that food packaging manufacturers must face is reducing their environmental impact, which can be addressed through the shift from unnecessary plastic packaging to the production of bio-based biodegradable materials from agro-food wastes [1]. This transition from a linear to a circular economy model is encouraged by the Packaging and Packaging Waste Regulation [2], which establishes requirements for the packaging life cycle regarding both its reuse and the use of more eco-sustainable packaging alternatives.

Bio-based packaging materials are proposed as sustainable alternatives to plastics; the global production of plastic amounted to 381 million tons in 2015, and packaging accounts for 40% of plastic produced globally [3], thus creating an important environmental issue due to the depletion of natural resources and emission of greenhouse gasses into the atmosphere. The contribution of the food industry to plastic consumption is notable: in Europe, 40% of food is packed into plastic containers [4]. The use of agri-food wastes in the production of biodegradable packaging materials could also solve the issues arising from their disposal, since every year, 1.3 billion tons of food waste are generated worldwide [5]. Although food waste/loss occurs at every stage across the food supply chain from production to consumption, 24% occurs in the production stage, and more than 24% occurs in postharvest operations [6]. Most food waste is intended for animal feed, but some of these materials could be conveniently used in the development of bioplastics as a valuable alternative to plastics derived from petroleum.

Several challenges arise in the production of bio-based packaging materials. The first is their poor mechanical and barrier properties when compared to synthetic plastics. However, they can be blended and/or reinforced with other materials or subjected to treatments suitable for overcoming these issues without compromising their biodegradability [7]. Another issue concerns the availability and seasonality of waste and by-products, which require infrastructure for their collection and processing [8,9]. Another challenge is the production of naturally biodegradable materials since most currently produced biodegradable plastics do not decompose in a reasonable time under environmental conditions of temperature and humidity, with serious concerns for fauna and flora [10]. Finally, a barrier to waste valorization through the development of bio-based materials is represented by the absence of clear, comprehensive, and supportive regulations and standards [9]. However, the production of a regulatory framework could be accelerated by the growing demand for bio-based polymers (biodegradable or not) in the European market, whose CAGR (Compound Annual Growth Rate) is around 21% [11].

Starch has received considerable attention from researchers involved in thermoplastic film production because it is a renewable raw material, abundantly available at low cost, and biodegradable [12]. Corn is the most important commercial starch source [13]. Among the most promising agro-industrial by-products, a prominent place is occupied by brewers’ spent grain (BSG), generated by the global brewing industry to the extent of 38 million tons [14]. Beyond being generated in massive quantities, BSG is quite inexpensive, having an estimated market value of € 35–50 per ton, and lacks cost-effective applications [15], as 70% is currently used as animal feed and 10% to produce biogas. The remaining 20% is disposed of in landfills and submitted to incineration procedures [16], significantly contributing to waste issues deriving from land usage and methane emissions.

In terms of dry matter, the chemical composition of BSG is as follows: 19–41.3% hemicellulose, with arabinoxylans as the main components, ranging from 2.7 to 21.9%; 15.2–28.7% cellulose; 3.35–21% lignin; 18.5–24.7% proteins; 8.4% lipids; 5.3% starch; 3.7% ash; and 0.7–2% vitamins and phenolic compounds [17,18]. This composition makes BSG suitable for biofilm production. In fact, arabinoxylans give the material good thermal stability [19] and, being soluble polysaccharides, they can form emulsions with high viscosity and gelling capacity [20]. BSG protein fractions are also usable in the production of active food packaging with desirable mechanical properties, water resistance, and antioxidant capacity [21]. The theoretical basis for selecting BSG as a component of thermoplastic starch films depends on both its chemical composition and structural compatibility with starch-based polymers. In fact, BSG contains cellulose, hemicellulose, lignin, and proteins, which behave like reinforcing fibers. Moreover, both thermoplastic starch and BSG components (especially cellulose and hemicellulose) are rich in hydroxyl (-OH) groups, thus allowing physical interaction via hydrogen bonding [22]. Most research has focused on bio-based composite films produced with proteins or arabinoxylans, or phenolic compounds extracted from BSG [19,21,23,24]. However, few studies are available on the development of thermoplastic films produced using dried and sieved BSG as it is. As an example, Castanho et al. [22] developed thermoplastic corn starch films filled with BSG. They tested three levels of dried BSG content (1, 2.5, and 5 wt.%) and three different sieves (35-, 60-, and 100-mesh) and evaluated the mechanical, morphological, and thermal properties of the developed films. Gelatinization was induced by microwave heating at 400 W for 7 min. The most promising films in terms of mechanical properties were produced with 2.5 wt.% of BSG-35-mesh sieve, and with 1 wt.% of BSG-100-mesh sieve. The better performances of films produced with low amounts of small BSG fibers compared to those produced with high amounts of large BSG fibers were attributed to their higher surface area-to-volume ratio. In a recent study by Mendes et al. [25], BSG fibers (5 or 10 wt.%) were incorporated into a quaternary nanocomposite film produced by casting with cassava starch (8% *w*/*v*), cocoa butter (30 wt.%), and lemongrass essential oil. BSG incorporation reduced lightness, thermal stability, tensile strength, and water vapor transmission rate, and increased thickness and extensibility. These changes occurred because the BSG fibers created a dispersed phase, thus restricting the movement of biopolymer chains. Hejna et al. [26] tried to improve the interfacial compatibility of BSG with Mater B polymer matrix by modifying BSG fibers through thermomechanical and chemical treatments before their use as filler. The thermomechanical modification occurred in a twin-screw extruder at temperatures of 180 or 240 °C, while the chemical modification occurred in a treatment with isophorone diisocyanate. The thermomechanical treatment stimulated the occurrence of caramelization and Maillard reactions thus increasing the antioxidant activity of filler while the chemical treatment increased thermal stability and compatibility of the composite material.

The low density of many starch–biocomposite films processed by extrusion makes them brittle and rigid [27]. Quinez-Molina et al. [28] used microwave (MW) irradiation to produce expanded starch films with low density values and exceptional flexibility. Li et al. [29] applied ultrasound treatments to increase the extraction yield of protein from BSG. They observed that ultrasonication was also able to modify protein BSG structure, resulting in increases in free sulfhydryl (SH) content and the proportion of the β-sheet, and decreases in α-helix, β-turn, and random coil structures. The exposure of interior SH groups and reduction in α-helix content indicated that the protein stretched, becoming loose and increasing softness and flexibility. However, it has never been verified whether this side effect on protein structure also occurs when ultrasonication is applied to thermoplastic starch–BSG composite formulations as a preliminary treatment to gelatinization.

Using these findings as a starting point, our research aimed to develop thermoplastic starch–BSG composite films. The effects of different BSG amounts (0, 1, 3, 5%), an ultrasonication pre-treatment, and different microwave gelatinization treatments (450 W for 80 and 90 s; 900 W for 45 and 50 s) on the physical and structural properties of the films were investigated to identify the most promising film compositions in terms of both degradability and possible uses. Compared to existing studies, the novelty of combining ultrasonication and microwave treatment in thermoplastic starch–BSG films is the synergistic integration of two phenomena: the dispersion of BSG fibers and the improved interfacial adhesion induced by ultrasound (at the physical level), and molecular restructuring, i.e., a combination of starch gelatinization, plasticization, starch–fiber interactions determined by microwaves (at the chemical level). This combination could enhance interfacial compatibility, thus creating a more homogeneous and compact film network that could significantly affect film performance.

## 2. Materials and Methods

### 2.1. Materials

#### 2.1.1. Characteristics and Treatment of BSG

The BSG used in our experiment was a by-product of Belgian-style white beer brewed from a mixture of 65% malted barley and 35% unmalted common wheat. BSG was dried at 20 °C for 20 ± 2 h (final moisture of 4.2 ± 0.2%) using an in-house-built forced-air drying system consisting of a fan placed under a stainless-steel grid hosting the wet BSG grains arranged in a thin layer (2 ± 0.5 cm). The dried BSG had the following characteristics: ash, 3.0 ± 0.1%; soluble and insoluble dietary fibers, 2.1 ± 0.0% and 25.2 ± 0.1%, respectively. The dried BSG was milled through a 2500 g Electric Grain Mill Grinder (VEVOR, Shanghai, China). The final particle size distribution, determined by an AS300 vibrating sieve shaker (Retsch Italia, Pedrengo, Italy), was as follows: <15%, >500 μm; 35–45%, 500–250 μm; 30–40%, 250–125 μm; <15%, <125 μm.

#### 2.1.2. Other Ingredients

Corn starch was produced by Cameo SPA (Desenzano del Garda, Italy). Glycerol of vegetable origin compliant with the pharmacopoeia and suitable for food and cosmetic use (G) was supplied by Farmacondo (Valeggio sul Mincio, Italy).

### 2.2. Production of the Thermoplastic Starch–BSG Composite Films

The formulations, producing conditions, and experimental plan described in this work were decided based on the results of preliminary tests.

The composite film formulations are shown in Table 1. The composite films were prepared with distilled water, glycerol, corn starch, and increasing BSG content. The amount of corn starch was reduced proportionally to the increase in BSG to keep the weight of the powdered ingredients constant. The film preparation method is the result of several preliminary ingredient mixing tests. In the method chosen for the tests, the required amount of starch was added to the required amount of distilled water and mixed by magnetic stirring on a stirring plate at 350 rpm for 15 min. Similarly, the BSG was added to the glycerol and subjected to magnetic stirring on a stirring plate at 350 rpm for 15 min. Finally, the two suspensions were mixed, again on a stirring plate at 350 rpm for 15 min.

**Figure 1:**
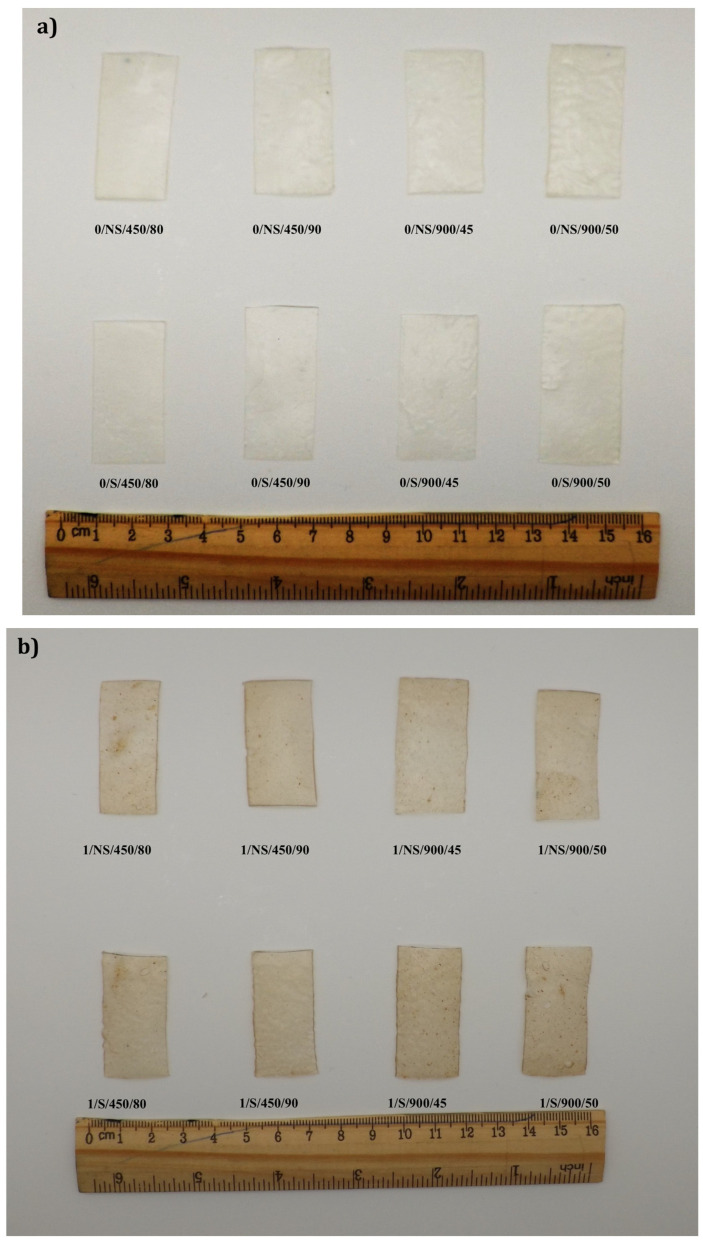

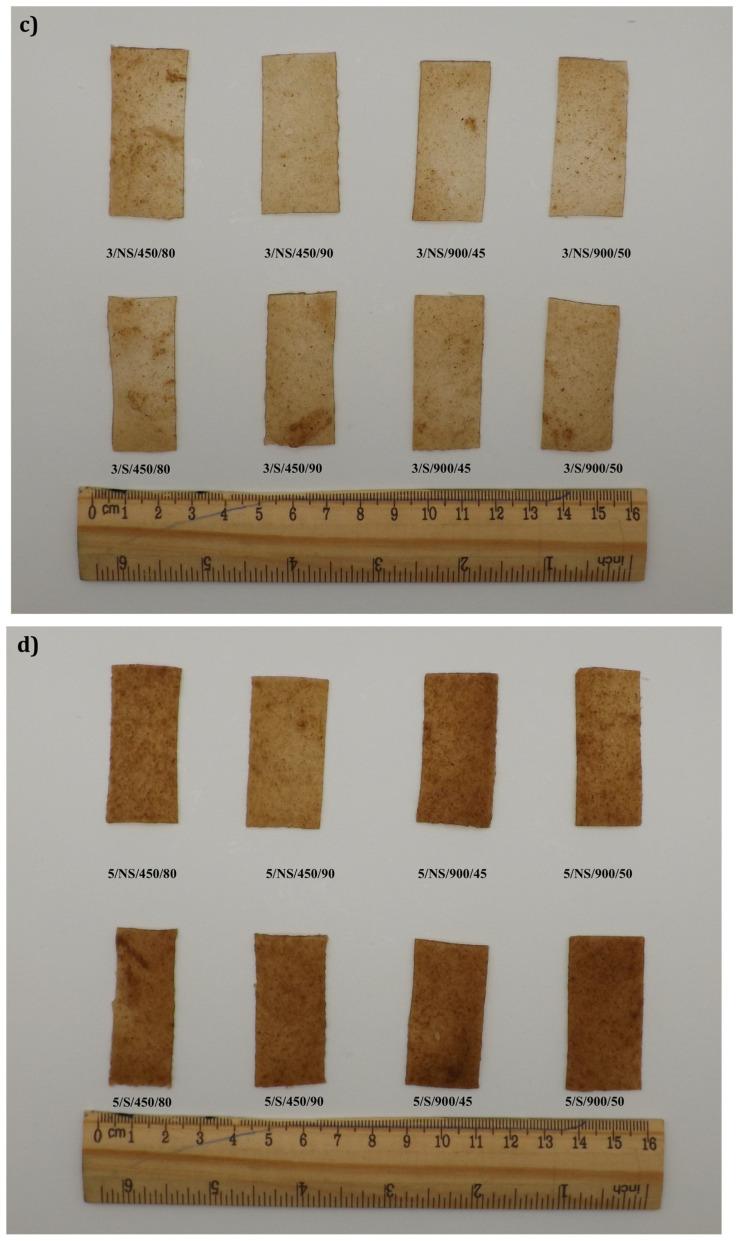
Thermoplastic composite films produced (**a**) without BSG, (**b**) with 1% BSG, (**c**) with 3% BSG, and (**d**) with 5% BSG.

Table 2 describes the comprehensive experimental plan. Thirty-two types of films (Figure 1) were prepared by combining the following three factors: BSG content (0, 1, 3, 5%); sonication [not sonicated; sonicated at 55 °C, 15 min, 40 KHz, 120 W; and gelatinization through microwave heating, performed according to the 4 different combinations of power and time of treatment: 450 W/80 s; 450 W/90 s; 900 W/45 s; 900 W/50 s. The samples without BSG were the references. The tested BSG concentrations were selected based on the results obtained from preliminary tests. During these tests, higher concentrations were also tested, but with negative effects on the mechanical properties of the films, which were found to be more brittle.

Regarding film preparation, corn starch was added to distilled water and mixed by magnetic stirring at 350 rpm for 15 min. BSG was added to glycerol and mixed by magnetic stirring again at 350 rpm for 15 min. The two suspensions were mixed by magnetic stirring at 350 rpm for 15 min to give the final film mixture. Half of the resulting mixture was directly subjected to the microwave gelatinization described, while the other half was previously subjected to ultrasonic treatment in a digital Ultrasonic Processor with 1 transducer (model DU-32 ArgoLab, Carpi, Italy) to improve the mixture’s homogeneity. For the microwave gelatinization, 30 g of the mixture was placed in a 100 mL Pyrex beaker (model AG925CTW, Kennex, Hong Kong, China; 230 V~50 Hz; input power, 1450 W; output power, 900 W; microwave frequency, 2450 MHz), chosen because it is faster and occurs through a different (asynchronous) mechanism with respect to that occurring in samples heated by using conduction heating, thus affecting the resulting gel’s properties [30]. The gel temperature on removal from the microwave oven was between 90 and 95 °C. Each type of film was produced in triplicate.

The hot gels were poured into 2 mm thick molds. A gentle pressure was applied through counter-molds with a diameter larger than that of the mold to remove the excess gel. After approximately 30 min, the counter-molds were removed, and the samples were dried in a food desiccator (Royal Catering AAA0000236962, Berlin, Germany) at 30 °C for 30 h. The films were then removed from the molds and stored at room temperature in desiccators containing granular silica gel.

### 2.3. Characterization of Starch–BSG Composite Films

#### 2.3.1. Scanning Electron Microscope Analysis (SEM)

The surface (approximately 10 mm × 10 mm)and cross-sections of film samples, as well as corn starch and BSG, were observed using a Hitachi TM3030 Tabletop scanning electron microscope (Tokyo, Japan) equipped with a digital camera. Samples were analyzed without coating and mounted on SEM stubs using conductive carbon adhesive tabs. The acceleration voltage was 15 kV with the following magnification values: 150×, 300×, 500×, and 600× for BSG flour; 2000×, 3000×, 5000×, and 7000× for corn starch; 600× and 1000× for the films.

#### 2.3.2. Moisture Content

Briefly, 1.5 g of each sample was dried in an oven at 105 °C for 24 h, cooled in a desiccator, and then weighed again. The moisture content was calculated according to Equation (1):

(1) $$Moisture\;content\;\%=\frac{\left(mi-mf\right)}{mi}\times 100$$

where *mi* is the mass of the wet sample, and *mf* is the mass of the dried sample. The analysis was performed in triplicate for each sample.

#### 2.3.3. Film Thickness

Film thickness was measured using a digital electronic caliper (model PRO-MAX, Fowler, Canton, MA, USA) with a resolution of 0.01 mm and an accuracy of 0.03 mm as the mean value of 10 measurements [31] and expressed as mm.

#### 2.3.4. Film Density

Five samples (2 cm × 2 cm) from each film were dried in a desiccator containing silica gel for approximately 5 weeks. The weight and volume of the samples were determined, and density was expressed as g/cm^3^ [32].

#### 2.3.5. Colorimetric Analyses

The L* (lightness), a* (position of color between red and green), and b* color (position of color between yellow and blue) coordinates of the experimental films were determined on 10 samples from each film using a Chroma Meter CR-400 (Minolta Camera Co., Ltd., Osaka, Japan) previously calibrated on a white standard plate. Chroma (measure of the vividness of a color, judged relative to an achromatic grey of the same lightness/value), hue (degree to which a stimulus can be described as similar to or different from stimuli such as red, orange, yellow, green, blue, or violet), ΔE (total color difference between the sample and a standard), and browning index (BI, degree of brown pigment formation) were determined according to Equations (2)–(5) [26]:

Chroma = [(a*)^2^ + (b*)^2^]^0.5^ (2)

Hue = arctan(b*/a*)(3)

ΔE = [(ΔL*)^2^ + (Δa*)^2^ + (Δb*)^2^]^0.5^ (4)

BI = [(x − 0.3)100]/0.17(5)

where x = (a* + 1.75L*)/(5.645L* + a* − 0.3012b*).

#### 2.3.6. Analysis of Transmittance Spectra for Determination of Optical Properties

Three samples (8 mm × 35 mm) from each film were analyzed. Each sample was fixed on the internal side of a quartz cuvette and transmittance spectra were recorded in the 200–800 nm range with an UV-21 spectrophotometer (Onda, Carpi, Italy). Ultraviolet (UV) radiation blocking was calculated in the UV_A (315–400 nm) and UV_B (280–315 nm) regions according to Equations (6) and (7) [33]:

UV_A blocking (%) = 100 − TUV_A(6)

UV_B blocking (%) = 100 − TUV_B(7)

where TUV_A and TUV_B are the average transmittance values in the corresponding spectral regions. Data refer to a 0.7 mm thick film.

Opacity was determined as described by Zhao et al. [34] according to (8):

Opacity = A_600_/film thickness (mm) (8)

where A_600_ is the absorbance at 600 nm.

#### 2.3.7. Determination of Water Solubility and Water Absorption

For the water solubility determination, three samples (2 cm × 2 cm) from each film were dried in an oven at 105 °C for 24 h, cooled to room temperature in a desiccator, and weighed. The dried samples were soaked in 10 mL of distilled water at room temperature for 24 h. After this time, samples were withdrawn, dried in an oven at 105 °C for 24 h and weighed. The water solubility of the samples was then calculated using Equation (9):

Water solubility % = [(w_i_ − w_fd_)/w_i_]100 (9)

where

w_i_ is the initial weight of the dried sample;

w_fd_ is the weight of the dried sample after being immersed in distilled water.

For water absorption determination [35], three samples (2 cm × 2 cm) from each film, previously dried at 105 °C for 2 h, were weighed and then immersed in distilled water at room temperature for 24 h. The weight of the wet sample was measured after removal of water from the surface with blotting paper. Percentage of swelling was calculated according to Equation (10):

Water absorption % = [(w_f_ − w_f_)/w_i_]100 (10)

where

w_i_ is the initial weight of the dried sample;

w_f_ is the weight of the wet sample after being immersed in distilled water.

#### 2.3.8. Soil Burial Degradation Tests

These tests were performed according to the method described by Lopes et al. [36] with some modifications. Briefly, samples dried in an oven at 60 °C until reaching a constant weight were cut (10 mm × 10 mm), weighed (t0), placed between two nylon meshes, and buried at 2 cm depth in 80 mL plastic containers containing 20 g of natural soil (Universal soil quality, COMPO SANA, Ravenna, Italy). The soil was made of neutral sphagnum peat moss, green compost soil improver, expanded perlite (less than 5%), and mineral fertilizer, and had the following characteristics: pH, 6.5; electrical conductivity, 0.50 dS/m; bulk density, 150 kg/m^3^; total porosity, 90% (*v*/*v*). The containers were kept at 24 °C. Every two days, 10 mL of water was added to maintain the soil moisture at approximately 50%. The degradation process was monitored by analyzing the samples after 3, 7, 10, and 14 days. At each time point, triplicate samples were carefully removed, visually inspected, weighed, and photographed to document the degradation progress. Samples were dried in an oven at 60 °C until a constant weight was reached, and their degradation (weight loss) percentage was calculated using Equation (11):

Degradation or weight loss (%) = [(w_bd_ − w_ad_)/w_bd_]100 (11)

where

w_bd_ is the initial weight taken before degradation;

w_ad_ is the final weight taken after 3, 7, 10, or 14 days of degradation.

#### 2.3.9. Tensile Strength Test

The film tensile properties [37] were measured using a 10 kN Allround Tisch Zwick Roell testing machine (Zwick/Roell, Ulm-Einsingen, Germany) equipped with fixed clamps rigidly attached to the fixed and movable members of the testing machine and with the TextXpert II software. Ten samples (sample size 2 cm × 4 cm) for each film type were conditioned at 23 ± 2 °C and 50% relative humidity for 10 days before analysis. The initial distance between clamps was fixed at 25 mm ± 1 mm, and the load interval was selected such that the maximum load occurred between 10% and 90% of the full-load scale. The specimen was assembled in the clamps such that the longest side was centered [38]. The analyses were performed according to a clamp separation rate of 5 mm/min. The following parameters were determined: Young’s modulus (N/mm^2^); tensile strength (N/mm^2^); and strain at maximum force (%).

#### 2.3.10. Statistical Analyses

Results were expressed as mean ± standard deviation. A three-way ANOVA followed by LSD test (*p* < 0.05) was applied to evaluate the effects of BSG contents, possible sonication, and microwave heating on each analytical parameter and the statistically significant differences among the film types.

The principal component analysis (PCA) was applied to verify relationships among the film types and the corresponding physio-chemical indices.

The response variables were fitted to the following second-order polynomial model:

(12) $$Y=\beta_{0}+\sum_{ì=1}^{k}\beta_{i}X_{i}+\sum_{ì=1}^{k}\beta_{ii}X_{ii}^{2}+\sum_{i}^{k-1}\sum_{j}^{k}\beta_{ij}X_{i}X_{j}$$

where Y is the response; β_0_ is the constant; β_i_ are the linear coefficients; β_ii_ are the quadratic coefficients; and β_ij_ are the interactive coefficients. ANOVA was used to evaluate the quality of the fitted model and to individuate the significant factors (*p* < 0.05), while the overall predictive capability of the model was evaluated by the coefficient of determination (R^2^). Response surface plots were generated. The statistical analyses were performed through Statistica for Windows V. 7.0 (Statsoft, Tulsa, OK, USA).

## 3. Results and Discussion

### 3.1. SEM Images

**Figure 2:**
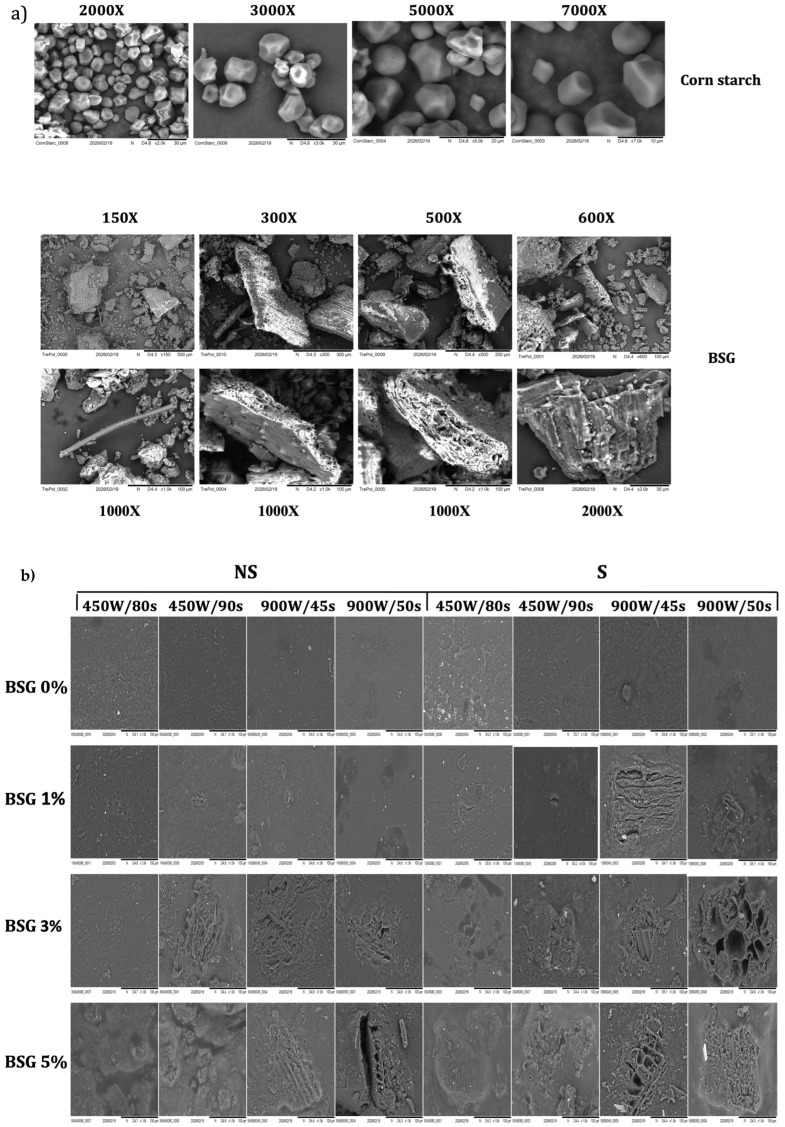

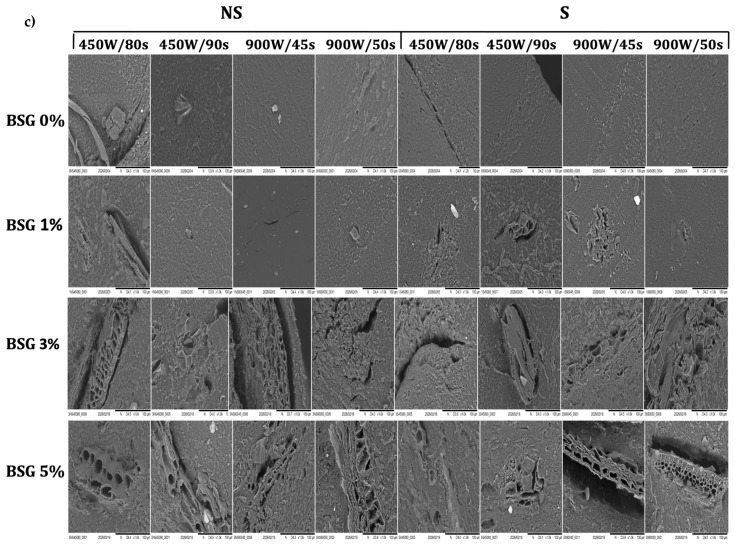
SEM micrographs of (**a**) corn starch and brewers’ spent grain; (**b**) film surfaces; (**c**) film cross-sections.

Scanning electron microscopy (SEM) was performed to investigate the microstructural features of the developed films and to support the interpretation of their physicochemical properties. Representative micrographs of native corn starch, brewers’ spent grain (BSG), and film samples are shown in Figure 2.

Figure 2a shows SEM micrographs of native corn starch and brewers’ spent grain (BSG) flour. Native corn starch granules exhibit the typical polyhedral morphology with smooth and compact surfaces, consistent with previous observations for ungelatinized starch systems [39]. In contrast, BSG particles display an irregular, rough surface composed of laminar fragments and a highly porous architecture [40]. This morphology reflects its lignocellulosic composition and explains its high water-binding capacity, as well as its role as a discontinuous phase when incorporated into starch-based matrices.

The surface morphology of the films (Figure 2b) is strongly affected by BSG content. Films without BSG show a smooth and compact surface, indicating the formation of a continuous and homogeneous thermoplastic starch network. With increasing BSG content, the surface becomes progressively rougher and more heterogeneous, with the appearance of pores and irregularities. This behavior suggests reduced compatibility between starch and BSG components and a less efficient embedding of the lignocellulosic particles within the polymer matrix. At 3% BSG, the presence of fiber agglomerates becomes evident, indicating partial phase separation and limited interfacial adhesion, which are known to reduce stress transfer efficiency in composite systems [41]. At 5% BSG, these effects are amplified, with clear evidence of structural discontinuities and surface fractures, confirming that high filler (BSG) loading compromises matrix integrity.

Sonication influences film morphology by modifying both particle dispersion and matrix organization. As observed in Figure 2b, sonicated samples generally exhibit smoother and more uniform surfaces, indicating improved dispersion of BSG particles. However, the presence of cavities and micro-voids suggests that cavitation phenomena induce local structural rearrangements [42]. In addition, as reported in the literature, fragmented particles may re-agglomerate due to increased surface reactivity, contributing to microstructural heterogeneity [43].

Microwave treatments further affect the film structure by promoting matrix consolidation. Increasing treatment severity results in more compact and cohesive structures, likely due to rapid volumetric heating, enhanced starch gelatinization, and accelerated water evaporation [30]. However, at higher BSG contents, the effectiveness of microwave-induced densification is limited by BSG fiber aggregation and poor interfacial compatibility.

Cross-sectional micrographs (Figure 2c) further confirm the influence of BSG incorporation on the internal structure of the films. Samples containing 3% and 5% BSG show clearly distinguishable fibrous domains and tubular voids associated with barley husk residues [44]. These features indicate incomplete integration of BSG within the starch matrix and the development of a multi-phase structure with poor interfacial continuity. The presence of voids and layered arrangements suggests limited cohesion between phases and contributes to the increased porosity and reduced mechanical performance of the films. Notably, the appearance of fractures at only 3% BSG indicates that the structural integrity of the matrix is compromised at relatively low filler contents.

Overall, SEM observations demonstrate that BSG content is the main factor governing film microstructure, while sonication and microwave treatments modulate particle dispersion and matrix consolidation. Low BSG levels allow relatively uniform structures, whereas higher contents lead to aggregation, porosity, and phase separation. These microstructural features are consistent with the observed trends in mechanical properties, water affinity, and optical behavior, confirming the strong relationship between morphology and functional performance in starch–BSG composite films.

### 3.2. Physical Characteristics of the Films

The physical properties of the films, including moisture content, thickness, and density, are reported in Table 3. These properties are closely related to the microstructural features highlighted by SEM analysis (Figure 2).

Film moisture content significantly increases with BSG incorporation (particularly at 3–5% levels), decreased with sonication, and decreased as the severity of microwave treatment increased (increasing power and/or time). All these changes are attributable to content and modifications undergone by the main components of BSG: hemicelluloses, celluloses, and proteins. Concerning the effects of BSG level, the increasing moisture content can be attributed to the hydrophilic nature of lignocellulosic components, which introduce a higher number of hydroxyl groups capable of binding water [45]. In addition, SEM observations reveal increased porosity and the presence of voids at higher BSG contents, which facilitate water retention within the matrix. Furthermore, protein–protein interactions and aggregation phenomena contribute to the formation of larger void spaces that can accommodate water molecules [22]. In addition, protein–starch interactions disrupt starch chain packing and create a more heterogeneous network, facilitating water incorporation [46]. The negative effect of sonication can be explained by structural modifications of BSG proteins. Ultrasonic treatment promotes partial unfolding of protein structures and exposure of hydrophobic groups, reducing the overall affinity of the matrix for water [29]. This effect is consistent with SEM observations showing improved dispersion and a more homogeneous microstructure in sonicated films. The effects of microwave treatments can be attributed to rapid internal heating and enhanced water evaporation, as well as to increased intermolecular interactions within the matrix [30,47]. SEM images support this interpretation, showing a more compact and cohesive structure at higher microwave intensities, with reduced porosity and fewer voids available for water retention.

Film thickness and density are also influenced by BSG content and processing conditions. Thickness increases with BSG content, likely due to reduced shrinkage during drying because of higher water retention and structural heterogeneity. In contrast, density increases with microwave treatment severity, reflecting the formation of a tighter and more consolidated network structure. This trend is consistent with SEM observations showing progressive densification of the matrix at higher microwave power and treatment time.

According to these findings, the films’ physical properties seem to be governed by the interactions between composition and structural modifications induced by processing: the increase in BSG content promotes porosity and water retention, while microwave treatment enhances matrix densification and reduces moisture content.

### 3.3. Chromatic Properties

As can be seen from the data in Table 4, L* and Hue significantly decrease under the following conditions: as BSG content increases; in films obtained from previously sonicated gel; and under the microwave treatment carried out at the highest power. Under the same conditions, a*, b*, C*, ΔE, and BI show the opposite trend, highlighting increases in reddish, yellowish, and brown colors. The chromatic changes due to increasing levels of BSG changes are primarily attributed to their darker colors, but also to their content of amino acids and free reducing sugars, which are substrates of Maillard reactions during processing [48]. Sonication further enhances browning, as evidenced by higher BI values. This effect can be attributed to ultrasound-induced protein unfolding, which exposes reactive amino groups (e.g., lysine), thereby promoting Maillard reactions [20]. Microwave treatment influences chromatic properties depending on processing severity. At lower microwave intensity, pigment degradation prevails, corresponding to an increase in L* value and decreases in a* and b*. At higher power levels, the accumulation of Maillard reaction products is promoted by the higher temperatures reached, leading to darker films [49].

### 3.4. Optical Properties

**Figure 3:**
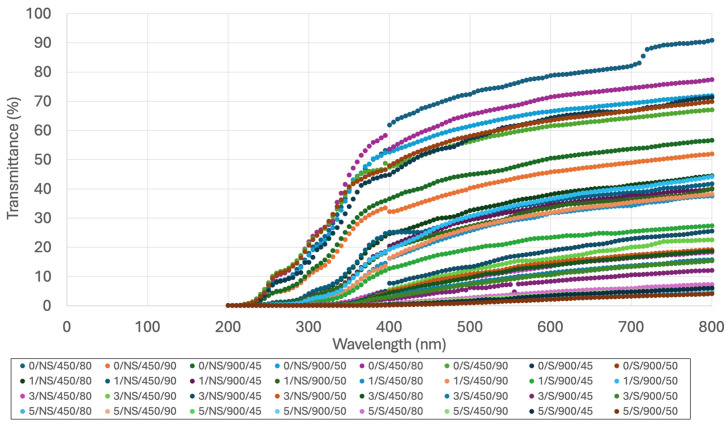
Transmittance of the thermoplastic starch–BSG composite films in the UV–visible range. Data refer to a standard 0.7 mm thick film.

The optical behavior of the films was evaluated through UV–Vis transmittance spectra in the 200–800 nm range (Figure 3) and related parameters reported in Table 5. As can be inferred from the curve trends, transmittance increases sharply in the UV region (200–400 nm), while it increases more gradually in the visible range, consistently with the typical behavior of starch-based films. Increased BSG content reduces film transmittance, particularly of visible light, due to its dark color and components like fiber and proteins, which decrease film transparency (see also Figure 1). As a result, opacity increases proportionally with BSG content, potentially limiting visual acceptability but enhancing light-barrier properties. The UV_A and UV_B blocking capacity of the film increase as the BSG content increases. This behavior is associated with the presence of aromatic amino acids and phenolic compounds in BSG, which act as natural UV absorbers due to their chromophoric structures [22]. However, BSG phenolic compounds also exert photoprotective effects due to their specialized chemical structures (chromophores) that absorb UV_A and UV_B radiation. In addition, SEM observations suggest interactions between starch, proteins, and fiber, which facilitate the uniform dispersion and stabilization of these chromophores within the matrix, contributing to effective UV attenuation [50]. Finally, the incorporation of BSG particles increases light scattering within the film matrix, further enhancing UV attenuation [51]. UV_A blocking capacity was higher than the UV_B blocking capacity, but the differences between the two values decreased as BSG content increased. Opacity is another optical property that quantifies the ability to prevent the passage of light through its section, consisting of a combination of light scattering and absorption. Opacity increased proportionally with BSG content. This aspect should be considered as it could limit film acceptance, making them visually less attractive [52].

Sonication increased UV blocking capacity since it can disrupt, break down, or modify these light-absorbing structures [53]. Sonication slightly reduces opacity, as supported by SEM analysis, which reveals a smoother and more homogeneous surface morphology. The fragmentation and improved dispersion of BSG particles reduce surface roughness and structural heterogeneity, thereby decreasing light scattering and resulting in lower opacity [54].

UV blocking capacity is increased by the two intermediate combinations of microwave treatment (low power–long time; high power intensity–short time), probably because they can induce the mechanical rupture of covalent bonds in specialized polymers, leading to the activation of embedded chromophores [55]. Microwave treatments reduce opacity as processing severity increase, probably by inducing BSG breakdowns and decreases in particle sizes.

To summarize, optical properties are governed by both chemical absorption (phenolics, proteins) and physical scattering effects (microstructure), which are strongly influenced by BSG content and processing conditions.

### 3.5. Water Resistance

Data concerning water absorption and solubility are presented in Table 6. Water absorption data highlight the same trends of film moisture, increasing as BSG content increased, decreasing with sonication, and decreasing as power and/or time of microwave treatment increased.

According to these results, starch–BSG composite films could be used as absorbent pads in the packaging of foods that produce exudates to make their appearance acceptable and attractive. Otherwise, the presence of BSG reduces film barrier properties against water penetration as well as the durability of the bio-composite material itself. Water uptake is also known to worsen the film’s mechanical properties, as described in Section 3.7. However, the introduction of an ultrasonic treatment in the production process, as well as the application of a more severe microwave heating, can increase water resistance, making the films less hydrophilic and hence less sensitive to the presence of water, even in the absence of a coating with waterproofing materials.

The water solubility of the films increases with an increasing amount of BSG due to an increase in film hydrophilicity. It also increases with sonication, probably due to partial depolymerization of matrix components and the release of low-molecular-weight compounds, such as sugars and amino acids, and polyphenols. This effect of sonication negatively affects the film’s water resistance, counterbalancing its capability to reduce water absorption. The effect of microwaves is less evident, and probably related only to the extension of treatment times, since a greater solubility was found only for films treated for 90 s.

These results indicate that processing conditions can partially compensate for the increased hydrophilicity induced by BSG, allowing modulation of water-related properties depending on the intended application.

### 3.6. Film Degradability

To evaluate the results of the soil degradation test, data concerning the weight loss at 3, 7, 10, and 14 days are reported in Table 6. The data shows that the degradation process is very rapid, since approximately 50–55% of the weight is lost in the first 3 days of burial, and the loss amounts to approximately 75–78% at the end of the experiment.

BSG content, sonication, and microwave treatment influence degradation kinetics depending on exposure time. At early stages (the first 3 days), higher BSG contents (3–5%) promote faster degradation due to increased hydrophilicity and porosity, which probably facilitate microbial activity. At later stages (after 14 days), films without BSG or with 1% BSG exhibit higher degradation rates, possibly due to their more homogeneous structure and easier enzymatic accessibility.

Films from sonicated gels show higher weight loss after 3, 7, and 10 days of burial; the opposite behavior was observed at 14 days. Regarding the effect of microwave treatment, films from gels treated at 900 W for 45 s have the highest weight loss after 3 days and the lowest values of this parameter during the other three samplings.

**Figure 4:**
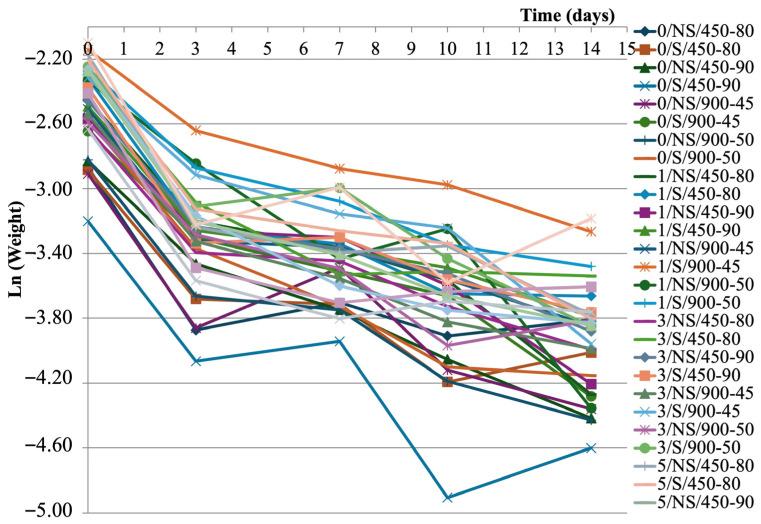
Kinetics of weight loss during soil burial degradation tests.

The modeling of weight loss kinetics has been performed. As can be seen in Figure 4, the natural logarithm of sample weight has been plotted versus time. The trendline of each film follows a first-order kinetic model:

ln(W_t_) = ln(W_0_) − kt (13)

where W_t_ is the weight at time t; W_0_ is the weight at time 0; t is the time; and k is the rate constant.

This model generally provides a good fit of organic waste degradation data in a natural soil environment [56]. The high value of the correlation coefficients (0.670 < R > 0.981 at *p* < 0.05) indicate that the model fitting is accurate.

During the soil degradation test, the erosion of polymers starts with their hydrolysis. Hydrolysis can be considered as a bimolecular reaction in which the speed of the process depends on the concentration of either reaction substrate. This would explain the higher slope (i.e., weight loss rate, k) of the early degradation steps [57]. However, the weight loss trends are influenced by other mechanisms of degradation, namely microbial, enzymatic, and chemical, which can occur [58,59]. The degradation rate is in the 0.08–0.09 g/day range for all the film samples.

### 3.7. Tensile Properties

The mechanical properties measured through tensile tests are reported in Table 7. They are strongly influenced by protein–starch–fiber interactions, which modify the structural integrity and load transfer within the matrix.

Young’s modulus is a property of solid materials that measures the tensile stiffness when the force is applied lengthwise. Tensile strength is the maximum stress to which a material can be subjected while being stretched or pulled before breaking. The considered factors affect the two parameters in the same way. In more detail, when added in low amounts (1%), BSG significantly increases the Young’s modulus and tensile strength of the starch film. This behavior is attributed to interaction among BSG components (proteins and fibers) and starch chains through hydrogen bonding and physical entanglement, leading to improved intermolecular cohesion and restricted polymer chain mobility [50,60]. This results in a reinforcing effect, enhancing stiffness and strength. Additionally, the good dispersion of BSG components at low concentrations promotes efficient stress transfer across the matrix [3]. Instead, a significant worsening of the mechanical properties was observed as BSG content increased due to increased heterogeneity and reduced interfacial compatibility. It can be assumed that the lignocellulosic fibers tend to aggregate, resulting in a weak adhesion between starch matrix and fiber particles and the appearance of discontinuities and stress concentration points [61]. Furthermore, excessive disruption of starch–starch interactions by proteins and fibers reduces matrix continuity, leading to lower tensile strength and modulus. Sonication significantly increases Young’s modulus and tensile strength. This change in mechanical properties may be due to the vibration induced by ultrasounds that allows water molecules to distribute homogeneously throughout the matrix [62]. Except for the treatment at 450 W for 90 s, the microwave treatments strongly increased Young’s modulus and tensile strength because of changes occurring in fiber structures that became well-ordered and homogeneous, and with a higher surface-area-to-volume ratio [63].

Strain at maximum force, also called “strain at ultimate tensile strength”, is the deformation recorded at the peak of a stress–strain curve. It marks the limit of uniform plastic deformation before necking occurs. It decreases as BSG content increases since fiber uptake makes the material brittle, especially in a homogeneous material, easily breaking or cracking if subjected to excessive stress or repeated bending [64]. These characteristics limit the scope of technological applications of the material to specific applications where fragility is an intrinsic characteristic of the functional material or where controlled breakage is desired. Sonication further decreases the strain at maximum force, resulting in a strong and brittle material because of ultrasound vibration [65].

### 3.8. Results of PCA and Predictive Capability of the Fitting Models

**Figure 5:**
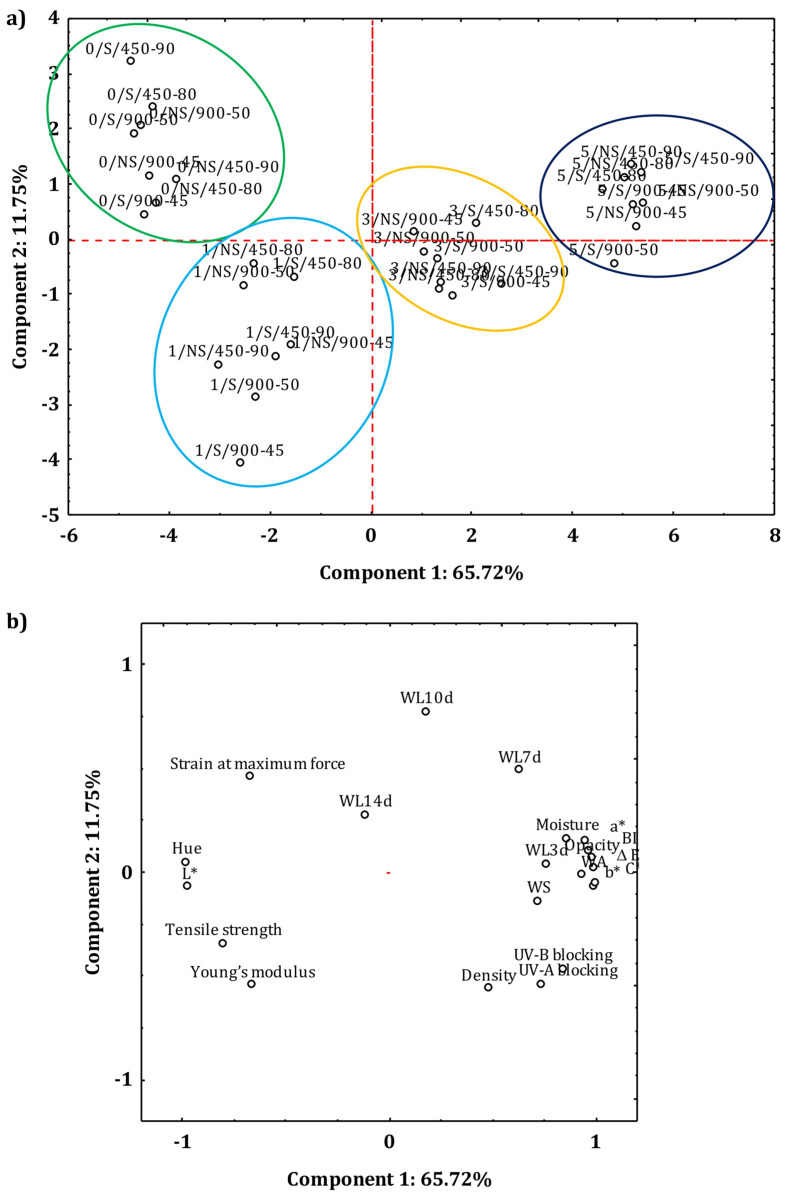
Principal component analysis: projection on the plane of (**a**) the film samples; (**b**) values of the corresponding variables.

Figure 5a,b show the distribution of film samples and dependent variables on the plane identified by the first two components, which can explain 77.47% of the total variance. This graphical representation allows one to choose the most suitable material for the intended use since it groups the film samples based on their chromatic, optical, water resistance, water solubility, and mechanical properties. Film samples are evenly distributed into four clearly defined groups based on their BSG content. The films without BSG are in the region of the plane identified by negative values of component 1 and positive values of component 2; they are characterized by the highest values of L*, Hue, and strain at maximum force. Samples containing 1% BSG are in the region identified by negative values of both components and have the highest Young’s modulus and tensile strength. Films produced with 5% BSG are in the section of the plane defined by positive values of both components (except for the 5/S/900-50 sample) and showed the highest values of moisture, density, a*, b*, C*, ΔE, browning index, UV blocking capacity, opacity, water absorption, water solubility, and weight loss at 3 and 7 days. The films with 3% BSG are in the area characterized by positive values of component 1, but astride the line passing through the 0 value of component 2.

Regression analysis was performed to create the equations able to describe the relationships between the three considered factors (BSG content, sonication, and microwave treatment) and each of the most important properties of the films. The equations can help to maximize or minimize a certain characteristic of the films, depending on their desired use. The ANOVA of the models with some statistical parameters (R^2^; F; SS, df, and MS of the models; residual SS, df, and MS) allowed the evaluation of the models’ ability to fit the data to a 95% confidence level (*p* < 0.05). The equations and the corresponding statistical information are reported in Table 8. Since only the statistically significant regressors (*p* < 0.05) are reported in the equations, the quadratic model can effectively describe the relationships of most properties with the three considered factors. The exceptions are parameters such as water solubility, weight loss at 3 days, and weight loss at 14 days, whose equations highlight linear relationships. The high F values (at *p* < 0.05) indicate that all models are significant. However, the models that better predict the variable behavior are those referred to the following parameters: UV_A blocking; UV_B blocking; water absorption; Young’s modulus; and tensile strength. The corresponding equations have R^2^ values higher than 0.6, and the variability explained by the model (model SS) is higher than 70% of the total variance (some of the model and residual SS).

According to the corresponding equations, UV_A and UV_B blocking capacity increase with BSG content, severity of microwave treatment, and conducting sonication (all as linear terms). Instead, the quadratic terms of both BSG content and microwave treatment, as well as the interactive effects of such parameters, decrease the ability to block UV_A and UV_B.

Water absorption is increased by BSG content (linear term) and by BSG content–microwave treatment interaction, while it is reduced by microwave treatment as a linear term, BSG content as a quadratic term, and microwave treatment–sonication interaction.

Young’s modulus increases with BSG content (as a linear term), microwave treatment (as a quadratic term), and the interaction between microwave treatment and sonication. Instead, it decreases with microwave treatment and sonication (both as linear terms), and as an effect of the interaction between BSG content and microwave treatment. These parameters exert the same effects on tensile strength but add the negative contribution of BSG content–sonication interaction.

To facilitate the graphic visualization of the effects of the considered factors, three iso-response surfaces have been built for each film property. They are shown in Supplementary Figure S1, together with the corresponding equations obtained by plotting two factors at a time, and keeping fixed the remaining factor at the central value of the experimental design (Supplementary Figure S1). All the properties are described by a quadratic fitting of BSG content and microwave treatment, and by the linear interactions of BSG content–sonication and microwave treatment–sonication.

### 3.9. Physicochemical Interpretation of Experimental Data and Statistics

The physicochemical behavior of the developed films can be interpreted as the result of the interactions between matrix composition and processing-induced structural reorganization. Increasing BSG content progressively transforms the system from a continuous thermoplastic starch matrix into a heterogeneous starch–fiber–protein composite. At low concentrations (1%), BSG acts as a reinforcing phase because cellulose, hemicellulose, and proteins establish hydrogen bonding and physical entanglements with starch chains, enhancing intermolecular cohesion and stress transfer, as previously reported for natural fiber–starch composites [50,60]. However, at higher BSG concentrations (3–5%), the system exceeds its compatibility threshold, leading to fiber agglomeration, poor interfacial adhesion, and the formation of structural discontinuities, as observed in similar lignocellulosic bio-composites [41,60]. Processing treatments further modulate these structural features. Sonication likely improves initial dispersion and promotes partial unfolding of protein structures, exposing reactive groups that enhance interfacial interactions during film formation, which contributes to the observed increase in stiffness and UV blocking capacity [29,62]. At the same time, cavitation-induced depolymerization and release of low-molecular-weight compounds explain the concurrent increase in water solubility [54]. Microwave treatment predominantly governs matrix consolidation through rapid volumetric heating, promoting starch gelatinization, increased intermolecular interactions, and water evaporation, resulting in denser and mechanically stronger films under more severe conditions [30,47].

The interactive effects among BSG content, sonication, and microwave treatment were statistically validated through the quadratic regression models and ANOVA (*p* < 0.05), confirming that not only main factors but also their interactions significantly influence film properties. The presence of interaction terms in the fitted equations and the high F values demonstrate that the effect of one factor depends on the level of the others. The significant interaction effects among BSG content, sonication, and microwave treatment indicate that processing efficiency is strongly composition-dependent: microwave-induced densification is more effective in starch-rich systems, whereas in highly BSG-filled systems it is limited by filler-induced heterogeneity; similarly, the benefits of sonication diminish at high BSG levels where filler–filler interactions dominate over matrix–filler adhesion.

In more detail, the BSG–microwave interaction is particularly relevant for structural and functional properties. For example, water absorption increases with BSG content but is partially mitigated by microwave treatment, as shown by the positive BSG term and negative MW term in the model, combined with a significant interaction coefficient. This indicates that microwave-induced restructuring (e.g., enhanced gelatinization and fiber–matrix bonding) counteracts the hydrophilic effect of BSG at higher energy inputs. Similarly, for mechanical properties, the negative BSG–microwave interaction suggests that although microwave treatment improves matrix cohesion, its reinforcing effect is less effective at high BSG levels due to fiber aggregation and reduced interfacial compatibility.

While sonication generally improves dispersion and mechanical performance, its interaction with BSG shows a negative contribution to tensile strength at higher BSG levels. This suggests that the structural modifications induced by ultrasound (e.g., protein unfolding and particle fragmentation) enhance compatibility at low BSG content but may promote aggregation or excessive matrix disruption when BSG concentration increases.

Finally, a synergistic effect between physical dispersion (sonication) and thermal restructuring (microwave treatments) is highlighted by the experimental data. For properties such as Young’s modulus, the positive interaction between sonication and microwave treatments indicates that combined treatments improve matrix homogeneity and interfacial adhesion more effectively than either treatment alone. This interaction also contributes to reduce water absorption, suggesting that the combined process produces a more compact and less permeable structure.

### 3.10. Practical Applications of the Experimental Films

As shown by the results, thermoplastic starch combined with BSG gave rise to biodegradable, flexible films with the potential to be used as eco-friendly alternatives for short-life cycle packaging and agricultural applications. They meet environmental standards for short-term packaging due to their high degradability. They can also be considered “home-compostable”, offering a significant reduction in long-term waste pollution. BSG increases polymer crystallinity, thus improving the thermal stability compared to pure thermoplastic starch.

Practical applications of such films include the following examples:

- Flexible food pouches/bags for dry goods such as snacks or cereals, where high moisture resistance is not required;
- Active edible coatings, since these materials could enhance food preservation due to the antioxidant properties of BSG phenolic compounds;
- Packaging for fresh goods, due to the inherent low barrier properties of these materials;
- Short-term food wrappers, to temporally protect them against potential contaminations from the environment;
- Disposable agricultural mulch films since they quickly degrade in soil.

## 4. Conclusions

The results obtained demonstrate the feasibility of producing flexible thermoplastic starch–brewers’ spent grain composite films characterized by high degradability. The addition of BSG determines a proportional increase in hydrophilicity, a characteristic already typical of starch-based films, making them more suitable for applications involving water absorption rather than for applications requiring a certain barrier effect of the film against water. Furthermore, while up to 1% spent grains improved the mechanical properties of the films (increased Young’s modulus and tensile strength), increasing the brewers’ spent grain concentration had a detrimental effect on the same parameters. Sonication and microwave treatments counteract the detrimental effect caused by the increased BSG content by reducing water absorption and improving mechanical properties. Sonication also contributes to increased UV blocking capacity. These results represent an important starting point for further research aimed at improving film performance by adding other ingredients to the formulation and/or through coating treatments. SEM micrographs highlight how the physical and mechanical properties of different films are related to their microstructure. The quadratic models were found to be satisfactorily capable of predicting some film characteristics based on variations in BSG content, possible sonication, and microwave treatment. In more detail, the best predictive ability was observed for properties related to UV blocking, water absorption, and mechanical properties.

The selected processing conditions are reproducible at laboratory scale under the condition that specific energy input and controlled sample geometry are guaranteed. While microwave treatments have good scalability potential when moving from a batch system to a continuous dielectric heating one, ultrasonication is more difficult to scale and requires process adaption because of a loss of efficiency due to the attenuation of cavitation effects at larger volumes. From a green perspective, the proposed approach is advantageous due to the avoidance of/reduction in chemical additives allowed by ultrasound and the low microwave processing time. Compared with other agro-waste-based films, the proposed materials represent a good compromise between functionality and scalability. Cellulose- and nanocellulose-based films generally provide superior mechanical and barrier performance, while protein-based films offer stronger gas-barrier potential. However, cellulose- and protein-based films often require more complex processing while the proposed films use minimally processed BSG as a low-cost filler and achieve excellent UV blocking and rapid biodegradability. Since moisture sensitivity and the loss of mechanical strength at higher BSG content limit their use in high-barrier applications, these thermoplastic starch–BSG films can be profitably employed for short-life, UV-protective, compostable packaging.

## Figures and Tables

**Table 1:** Formulations of the thermoplastic starch–BSG composite films (g/100 g).

Formulation	Ingredients			
	Distilled Water	Starch	Glycerol	BSG
0	80	10	10	0
1	80	9	10	1
3	80	7	10	3
5	80	5	10	5

**Table 2:** Experimental plan to produce 32 types of thermoplastic starch–BSG composite films.

Type	% BSG	Sonication	Microwave Heating
0/NS/450-80	0%	NS	450 W-80 s
0/S/450-80	0%	S	450 W-80 s
0/NS/450-90	0%	NS	450 W-90 s
0/S/450-90	0%	S	450 W-90 s
0/NS/900-45	0%	NS	900 W-45 s
0/S/900-45	0%	S	900 W-45 s
0/NS/900-50	0%	NS	900 W-50 s
0/S/900-50	0%	S	900 W-50 s
1/NS/450-80	1%	NS	450 W-80 s
1/S/450-80	1%	S	450 W-80 s
1/NS/450-90	1%	NS	450 W-90 s
1/S/450-90	1%	S	450 W-90 s
1/NS/900-45	1%	NS	900 W-45 s
1/S/900-45	1%	S	900 W-45 s
1/NS/900-50	1%	NS	900 W-50 s
1/S/900-50	1%	S	900 W-50 s
3/NS/450-80	3%	NS	450 W-80 s
3/S/450-80	3%	S	450 W-80 s
3/NS/450-90	3%	NS	450 W-90 s
3/S/450-90	3%	S	450 W-90 s
3/NS/900-45	3%	NS	900 W-45 s
3/S/900-45	3%	S	900 W-45 s
3/NS/900-50	3%	NS	900 W-50 s
3/S/900-50	3%	S	900 W-50 s
5/NS/450-80	5%	NS	450 W-80 s
5/S/450-80	5%	S	450 W-80 s
5/NS/450-90	5%	NS	450 W-90 s
5/S/450-90	5%	S	450 W-90 s
5/NS/900-45	5%	NS	900 W-45 s
5/S/900-45	5%	S	900 W-45 s
5/NS/900-50	5%	NS	900 W-50 s
5/S/900-50	5%	S	900 W-50 s

NS: not sonicated; S: sonicated.

**Table 3:** Moisture, thickness, and density of the thermoplastic starch–BSG composite films.

Type	Moisture (%)	Thickness (mm)	Density (g/cm^3^)
0/NS/450-80	12.07 ± 0.92	0.72 ± 0.10	0.893 ± 0.002
0/S/450-80	11.03 ± 0.28	0.69 ± 0.09	0.760 ± 0.000
0/NS/450-90	11.37 ± 0.80	0.74 ± 0.12	1.025 ± 0.001
0/S/450-90	10.22 ± 0.24	0.68 ± 0.10	0.705 ± 0.001
0/NS/900-45	11.07 ± 0.40	0.74 ± 0.09	0.859 ± 0.000
0/S/900-45	9.93 ± 0.34	0.72 ± 0.12	0.688 ± 0.001
0/NS/900-50	10.15 ± 0.17	0.75 ± 0.09	1.168 ± 0.000
0/S/900-50	9.95 ± 0.44	0.71 ± 0.12	1.219 ± 0.000
1/NS/450-80	11.28 ± 0.30	0.80 ± 0.09	0.995 ± 0.001
1/S/450-80	9.46 ± 0.33	0.81 ± 0.08	1.164 ± 0.001
1/NS/450-90	10.65 ± 0.84	0.73 ± 0.13	1.448 ± 0.001
1/S/450-90	10.12 ± 0.22	0.78 ± 0.08	1.186 ± 0.001
1/NS/900-45	9.51 ± 0.11	0.74 ± 0.11	1.456 ± 0.001
1/S/900-45	9.38 ± 0.17	0.85 ± 0.07	1.322 ± 0.001
1/NS/900-50	8.49 ± 0.77	0.79 ± 0.09	1.180 ± 0.001
1/S/900-50	9.12 ± 0.22	0.82 ± 0.07	1.303 ± 0.001
3/NS/450-80	20.23 ± 1.27	0.72 ± 0.12	1.503 ± 0.000
3/S/450-80	26.47 ± 2.01	0.78 ± 0.10	1.051 ± 0.000
3/NS/450-90	21.33 ± 0.45	0.77 ± 0.10	1.346 ± 0.000
3/S/450-90	24.40 ± 1.11	0.80 ± 0.11	1.274 ± 0.001
3/NS/900-45	23.35 ± 1.65	0.71 ± 0.13	1.170 ± 0.001
3/S/900-45	21.53 ± 0.72	0.79 ± 0.11	1.198 ± 0.000
3/NS/900-50	26.63 ± 1.80	0.72 ± 0.11	1.061 ± 0.001
3/S/900-50	17.11 ± 0.89	0.81 ± 0.08	1.154 ± 0.000
5/NS/450-80	20.49 ± 1.46	0.91 ± 0.07	1.046 ± 0.001
5/S/450-80	27.31 ± 4.22	0.87 ± 0.07	1.227 ± 0.000
5/NS/450-90	22.96 ± 2.06	0.86 ± 0.06	1.147 ± 0.001
5/S/450-90	25.28 ± 3.58	0.85 ± 0.09	1.361 ± 0.001
5/NS/900-45	25.60 ± 2.12	0.90 ± 0.06	1.375 ± 0.000
5/S/900-45	20.39 ± 2.88	0.89 ± 0.08	1.390 ± 0.001
5/NS/900-50	28.40 ± 0.82	0.83 ± 0.07	1.114 ± 0.001
5/S/900-50	16.77 ± 1.05	0.89 ± 0.10	1.492 ± 0.001
Effect of BSG Content			
0%	10.72 ^b^	0.72 ^a^	0.90 ^a^
1%	9.75 ^a^	0.79 ^c^	1.26 ^c^
3%	22.65 ^c^	0.76 ^b^	1.22 ^b^
5%	23.40 ^d^	0.88 ^d^	1.27 ^d^
Significance (*p* < 0.05)	*	*	*
Effect of Sonication			
Not Sonicated	17.08 ^b^	0.78 ^a^	1.16 ^a^
Sonicated	16.15 ^a^	0.80 ^a^	1.15 ^a^
Significance (*p* < 0.05)	*		
Effect of Microwave Treatment			
450 W-80 s	17.27 ^d^	0.79 ^a^	1.08 ^a^
450 W-90 s	17.04 ^c^	0.78 ^a^	1.17 ^b^
900 W-45 s	16.34 ^b^	0.79 ^a^	1.18 ^c^
900 W-50 s	15.83 ^a^	0.79 ^a^	1.21 ^d^
Significance (*p* < 0.05)	*		*

In column, different letters correspond to statistically significant values. * indicate significant differences within groups of samples.

**Table 4:** Chromatic properties of the thermoplastic starch–BSG composite films.

Type	L*	a*	b*	C*	Hue	ΔE*	Browning Index
0/NS/450-80	89.45 ± 1.36	−0.88 ± 0.13	6.05 ± 0.23	6.12 ± 0.23	98.27 ± 1.19	6.02 ± 1.30	6.12 ± 0.30
0/S/450-80	91.28 ± 0.78	−0.76 ± 0.05	6.02 ± 0.27	6.07 ± 0.27	97.18 ± 0.48	4.33 ± 0.80	6.05 ± 0.26
0/NS/450-90	90.83 ± 1.02	−0.78 ± 0.04	5.85 ± 0.26	5.90 ± 0.26	97.60 ± 0.36	4.67 ± 0.93	5.86 ± 0.28
0/S/450-90	91.78 ± 0.35	−0.71 ± 0.06	5.58 ± 0.25	5.65 ± 0.28	97.20 ± 0.68	3.69 ± 0.41	5.55 ± 0.30
0/NS/900-45	91.64 ± 0.20	−0.85 ± 0.04	6.05 ± 0.43	6.11 ± 0.43	98.05 ± 0.42	4.05 ± 0.33	5.98 ± 0.47
0/S/900-45	91.13 ± 0.61	−0.76 ± 0.05	5.56 ± 0.35	5.61 ± 0.35	97.77 ± 0.87	4.28 ± 0.52	5.54 ± 0.40
0/NS/900-50	91.20 ± 0.51	−0.82 ± 0.09	6.23 ± 0.31	6.28 ± 0.31	97.54 ± 0.60	4.52 ± 0.56	6.24 ± 0.34
0/S/900-50	91.86 ± 0.52	−0.74 ± 0.03	5.63 ± 0.26	5.68 ± 0.26	97.48 ± 0.38	3.63 ± 0.56	5.58 ± 0.31
1/NS/450-80	85.04 ± 2.31	−0.01 ± 0.00	14.04 ± 2.41	14.04 ± 2.41	90.24 ± 1.67	14.30 ± 3.26	17.80 ± 4.27
1/S/450-80	85.38 ± 0.54	−0.12 ± 0.06	13.77 ± 0.44	13.77 ± 0.44	90.50 ± 0.26	13.85 ± 0.67	17.08 ± 0.75
1/NS/450-90	87.36 ± 0.77	−0.38 ± 0.12	11.87 ± 0.79	11.88 ± 0.79	91.87 ± 0.33	11.10 ± 1.09	13.95 ± 1.17
1/S/450-90	86.12 ± 1.02	−0.18 ± 0.06	12.76 ± 0.83	12.76 ± 0.83	90.84 ± 0.58	12.60 ± 1.28	15.52 ± 1.37
1/NS/900-45	87.05 ± 1.22	−0.28 ± 0.09	11.96 ± 1.43	11.97 ± 1.43	91.42 ± 1.04	11.38 ± 1.87	14.23 ± 2.23
1/S/900-45	85.22 ± 1.57	0.04 ± 0.00	13.78 ± 1.81	13.78 ± 1.81	89.99 ± 1.46	13.98 ± 2.39	17.34 ± 3.08
1/NS/900-50	84.83 ± 1.75	0.03 ± 0.00	14.11 ± 1.71	14.11 ± 1.71	89.96 ± 0.71	14.48 ± 2.43	17.87 ± 3.01
1/S/900-50	84.90 ± 0.76	0.04 ± 0.00	13.74 ± 0.89	13.74 ± 0.89	89.87 ± 0.49	14.17 ± 1.15	17.29 ± 1.45
3/NS/450-80	76.12 ± 4.58	3.07 ± 1.41	22.96 ± 3.86	23.19 ± 3.99	82.75 ± 2.54	27.14 ± 6.08	38.98 ± 10.53
3/S/450-80	76.10 ± 2.46	2.84 ± 0.92	23.22 ± 1.81	23.41 ± 1.90	83.13 ± 1.73	27.31 ± 3.07	38.61 ± 5.59
3/NS/450-90	77.31 ± 2.94	2.33 ± 0.80	22.02 ± 2.63	22.15 ± 2.70	84.08 ± 1.30	25.55 ± 3.98	35.40 ± 7.16
3/S/450-90	75.00 ± 4.09	3.10 ± 1.25	23.57 ± 2.95	23.78 ± 3.08	82.72 ± 2.15	28.36 ± 5.06	40.62 ± 9.33
3/NS/900-45	78.26 ± 1.26	2.18 ± 0.43	21.30 ± 1.03	21.42 ± 1.06	84.18 ± 0.84	24.36 ± 1.57	33.20 ± 2.77
3/S/900-45	73.76 ± 2.21	3.39 ± 0.73	24.95 ± 1.62	25.05 ± 1.47	82.33 ± 1.11	30.22 ± 2.78	44.00 ± 5.72
3/NS/900-50	77.71 ± 3.30	2.56 ± 1.06	22.10 ± 2.90	22.25 ± 3.01	83.59 ± 1.65	25.35 ± 4.47	35.66 ± 8.54
3/S/900-50	73.99 ± 2.42	3.40 ± 0.84	24.65 ± 2.08	24.89 ± 2.17	82.25 ± 1.34	29.85 ± 3.24	43.32 ± 6.47
5/NS/450-80	57.39 ± 4.32	9.43 ± 1.37	31.04 ± 0.76	32.46 ± 0.96	73.14 ± 2.21	47.54 ± 3.87	87.35 ± 10.46
5/S/450-80	61.18 ± 2.39	8.29 ± 0.87	31.14 ± 0.88	32.23 ± 1.01	75.11 ± 1.26	44.39 ± 2.31	79.08 ± 6.96
5/NS/450-90	59.04 ± 3.43	8.79 ± 1.35	30.86 ± 0.77	32.10 ± 1.09	74.16 ± 1.99	45.98 ± 2.37	83.04 ± 10.10
5/S/450-90	60.39 ± 2.78	8.24 ± 0.86	30.48 ± 1.34	31.58 ± 1.40	74.88 ± 1.40	44.61 ± 2.41	78.37 ± 7.13
5/NS/900-45	54.71 ± 3.64	10.63 ± 1.30	30.74 ± 0.50	32.55 ± 0.55	70.93 ± 2.26	49.78 ± 3.15	93.29 ± 8.74
5/S/900-45	55.97 ± 4.36	10.19 ± 1.41	31.28 ± 1.02	32.92 ± 0.91	71.96 ± 2.62	48.99 ± 3.63	91.67 ± 9.05
5/NS/900-50	59.02 ± 3.91	9.11 ± 1.26	31.12 ± 0.60	32.44 ± 0.78	73.72 ± 2.03	46.23 ± 3.48	83.95 ± 9.56
5/S/900-50	55.93 ± 5.51	9.96 ± 1.96	30.42 ± 1.28	32.05 ± 1.55	71.95 ± 3.23	48.51 ± 5.13	90.26 ± 14.59
Effect of BSG Content							
0%	91.14 ^d^	−0.79 ^a^	5.87 ^a^	5.93 ^a^	97.64 ^d^	4.40 ^a^	5.86 ^a^
1%	85.74 ^c^	−0.11 ^b^	13.25 ^b^	13.26 ^b^	90.58 ^c^	13.23 ^b^	16.38 ^b^
3%	76.03 ^b^	2.86 ^c^	24.00 ^c^	23.27 ^c^	83.13 ^b^	27.27 ^c^	38.72 ^c^
5%	57.95 ^a^	9.33 ^d^	30.88 ^d^	32.29 ^d^	73.23 ^a^	47.00 ^d^	85.88 ^d^
Significance (*p* < 0.05)	*	*	*	*	*	*	*
Effect of Sonication							
Not Sonicated	77.94 ^b^	2.76 ^a^	18.00 ^a^	18.41 ^a^	86.36 ^b^	22.63 ^a^	36.17 ^a^
Sonicated	77.50 ^a^	2.89 ^b^	18.53 ^b^	18.93 ^b^	85.95 ^a^	23.30 ^b^	37.24 ^b^
Significance (*p* < 0.05)	*	*	*	*	*	*	*
Effect of Microwave Treatment							
450 W-80 s	77.76 ^b^	2.73 ^b^	18.49 ^c^	18.87 ^bc^	86.32 ^b^	23.07 ^b^	36.36 ^b^
450 W-90 s	78.48 ^c^	2.55 ^a^	17.87 ^a^	18.22 ^a^	86.67 ^c^	22.07 ^a^	34.79 ^a^
900 W-45 s	77.21 ^a^	3.07 ^d^	18.20 ^b^	18.68 ^b^	85.83 ^a^	23.38 ^b^	38.15 ^c^
900 W-50 s	77.49 ^ab^	2.94 ^c^	18.50 ^c^	18.93 ^c^	85.79 ^a^	23.34 ^b^	37.52 ^c^
Significance (*p* < 0.05)	*	*	*	*	*	*	*

In column, different letters correspond to statistically significant values. * indicate significant differences within groups of samples.

**Table 5:** Optical properties of the thermoplastic starch–BSG composite films.

Type	UV_A Blocking (%)	UV_B Blocking (%)	Opacity
0/NS/450-80	73.64 ± 17.89	59.10 ± 15.01	0.67 ± 0.11
0/S/450-80	82.82 ± 9.61	60.98 ± 16.68	0.53 ± 0.08
0/NS/450-90	90.38 ± 6.20	74.90 ± 11.12	0.53 ± 0.13
0/S/450-90	80.08 ± 9.33	60.26 ± 11.64	0.47 ± 0.07
0/NS/900-45	86.08 ± 5.92	67.50 ± 9.28	0.54 ± 0.17
0/S/900-45	85.36 ± 5.47	64.94 ± 7.84	0.42 ± 0.06
0/NS/900-50	80.46 ± 6.34	54.63 ± 10.36	0.45 ± 0.04
0/S/900-50	80.57 ± 7.93	60.55 ± 9.53	0.51 ± 0.05
1/NS/450-80	96.40 ± 3.46	85.65 ± 8.05	0.59 ± 0.09
1/S/450-80	98.68 ± 1.08	91.95 ± 4.09	0.65 ± 0.08
1/NS/450-90	96.39 ± 1.42	85.00 ± 3.99	0.68 ± 0.10
1/S/450-90	98.59 ± 0.37	92.11 ± 0.84	0.71 ± 0.09
1/NS/900-45	97.37 ± 1.74	88.65 ± 5.55	0.76 ± 0.14
1/S/900-45	99.06 ± 0.28	93.66 ± 1.46	0.68 ± 0.09
1/NS/900-50	97.46 ± 2.41	89.11 ± 7.74	0.60 ± 0.12
1/S/900-50	98.21 ± 0.67	89.46 ± 3.43	0.68 ± 0.14
3/NS/450-80	99.72 ± 0.16	97.58 ± 0.63	1.31 ± 0.03
3/S/450-80	99.86 ± 0.05	98.46 ± 0.62	1.14 ± 0.05
3/NS/450-90	99.85 ± 0.07	97.96 ± 0.75	1.16 ± 0.03
3/S/450-90	99.92 ± 0.03	99.07 ± 0.22	1.16 ± 0.05
3/NS/900-45	99.86 ± 0.05	97.86 ± 0.63	1.19 ± 0.11
3/S/900-45	99.95 ± 0.01	99.21 ± 0.13	1.14 ± 0.02
3/NS/900-50	99.87 ± 0.09	98.34 ± 0.99	1.09 ± 0.12
3/S/900-50	99.90 ± 0.03	98.80 ± 0.38	1.10 ± 0.10
5/NS/450-80	99.96 ± 0.01	99.84 ± 0.07	1.80 ± 0.08
5/S/450-80	99.96 ± 0.01	99.81 ± 0.15	1.65 ± 0.22
5/NS/450-90	99.96 ± 0.01	99.76 ± 0.16	1.69 ± 0.12
5/S/450-90	99.96 ± 0.01	99.83 ± 0.09	1.74 ± 0.09
5/NS/900-45	99.97 ± 0.01	99.91 ± 0.02	1.57 ± 0.09
5/S/900-45	99.95 ± 0.00	99.86 ± 0.04	1.58 ± 0.17
5/NS/900-50	99.97 ± 0.00	99.90 ± 0.02	1.48 ± 0.05
5/S/900-50	99.96 ± 0.01	99.92 ± 0.01	1.54 ± 0.04
Effect of BSG Content			
0%	82.42 ^a^	61.28 ^a^	0.51 ^a^
1%	97.77 ^b^	89.45 ^b^	0.67 ^b^
3%	99.87 ^c^	94.42 ^c^	1.16 ^c^
5%	99.96 ^c^	99.86 ^d^	1.63 ^d^
Significance (*p* < 0.05)	*	*	*
Effect of Sonication			
Not Sonicated	94.81 ^a^	86.40 ^a^	1.01 ^b^
Sonicated	95.18 ^b^	88.05 ^b^	0.98 ^a^
Significance (*p* < 0.05)	*	*	*
Effect of Microwave Treatment			
450 W-80 s	93.83 ^a^	84.99 ^a^	1.04 ^d^
450 W-90 s	95.64 ^c^	88.61 ^c^	1.02 ^c^
900 W-45 s	95.95 ^c^	88.95 ^c^	0.98 ^b^
900 W-50 s	94.95 ^b^	86.34 ^b^	0.93 ^a^
Significance (*p* < 0.05)	*	*	*

In column, different letters correspond to statistically significant values. * indicate significant differences within groups of samples.

**Table 6:** Water absorption, water solubility, and weight loss at 3, 7, 10, and 14 days of the thermoplastic starch–BSG composite films.

Type	Water Absorption (%)	Water Solubility (%)	Weight Loss at 3 Days (%)	Weight Loss at 7 Days (%)	Weight Loss at 10 Days (%)	Weight Loss at 14 Days (%)
0/NS/450-80	73.54 ± 7.89	42.69 ± 8.54	49.60 ± 4.25	55.92 ± 3.58	58.52 ± 13.25	68.59 ± 6.83
0/S/450-80	65.90 ± 2.53	43.57 ± 0.19	47.51 ± 25.59	59.16 ± 13.07	70.76 ± 20.03	71.64 ± 6.79
0/NS/450-90	73.45 ± 9.39	46.89 ± 2.80	41.92 ± 3.02	55.51 ± 0.18	71.56 ± 11.41	80.05 ± 9.42
0/S/450-90	62.03 ± 10.45	49.76 ± 4.05	45.07 ± 4.65	56.28 ± 8.56	79.46 ± 0.22	78.68 ± 5.03
0/NS/900-45	68.18 ± 7.45	43.29 ± 1.98	50.28 ± 2.90	52.70 ± 11.54	70.90 ± 9.15	76.56 ± 6.94
0/S/900-45	65.70 ± 4.51	50.58 ± 2.05	51.35 ± 2.04	56.29 ± 4.06	61.39 ± 0.87	80.97 ± 3.48
0/NS/900-50	70.64 ± 6.41	45.08 ± 0.53	53.24 ± 0.72	61.86 ± 1.44	72.21 ± 4.24	81.53 ± 4.02
0/S/900-50	61.78 ± 1.42	48.06 ± 0.52	53.85 ± 14.00	72.06 ± 3.38	76.42 ± 0.29	78.91 ± 8.19
1/NS/450-80	84.13 ± 4.68	55.16 ± 3.74	46.38 ± 3.99	52.55 ± 9.67	69.50 ± 10.52	84.84 ± 5.67
1/S/450-80	79.23 ± 1.62	50.75 ± 1.03	57.29 ± 1.29	60.89 ± 2.44	70.96 ± 19.03	77.41 ± 2.62
1/NS/450-90	67.11 ± 6.08	47.38 ± 7.54	44.01 ± 24.45	50.17 ± 3.91	61.31 ± 1.61	82.47 ± 2.72
1/S/450-90	76.76 ± 2.70	52.03 ± 1.33	54.47 ± 0.30	56.70 ± 3.59	62.04 ± 2.75	73.61 ± 3.36
1/NS/900-45	75.66 ± 4.27	50.75 ± 1.58	54.31 ± 2.13	56.70 ± 0.87	63.24 ± 4.61	76.74 ± 0.93
1/S/900-45	72.60 ± 4.02	49.42 ± 1.72	48.98 ± 3.45	49.13 ± 2.85	52.34 ± 0.82	66.50 ± 10.44
1/NS/900-50	72.55 ± 4.73	49.44 ± 1.19	38.46 ± 2.44	60.16 ± 5.26	66.51 ± 4.80	85.85 ± 5.90
1/S/900-50	69.58 ± 6.34	50.30 ± 1.69	53.15 ± 1.26	54.01 ± 0.17	63.70 ± 5.00	70.00 ± 6.75
3/NS/450-80	90.16 ± 5.73	47.17 ± 2.10	55.41 ± 0.48	56.57 ± 4.21	65.47 ± 2.99	73.26 ± 8.76
3/S/450-80	90.81 ± 7.09	50.76 ± 2.11	56.03 ± 4.89	64.53 ± 8.64	69.25 ± 6.24	74.53 ± 6.86
3/NS/450-90	92.89 ± 4.58	49.07 ± 2.08	57.07 ± 7.03	57.42 ± 0.47	62.72 ± 10.65	76.01 ± 4.09
3/S/450-90	85.87 ± 5.74	53.22 ± 1.73	62.92 ± 4.26	63.10 ± 9.21	63.81 ± 2.26	75.84 ± 2.89
3/NS/900-45	88.52 ± 7.15	47.02 ± 0.86	54.13 ± 2.65	62.74 ± 12.64	67.09 ± 9.32	78.52 ± 6.29
3/S/900-45	92.28 ± 7.46	52.64 ± 1.74	55.22 ± 1.34	60.70 ± 1.04	61.05 ± 2.94	76.38 ± 1.76
3/NS/900-50	92.89 ± 1.82	47.88 ± 1.89	54.15 ± 3.72	62.90 ± 3.10	62.87 ± 1.16	73.39 ± 4.03
3/S/900-50	91.91 ± 7.01	51.51 ± 0.99	53.99 ± 4.79	61.47 ± 5.61	66.26 ± 5.31	79.24 ± 6.40
5/NS/450-80	98.89 ± 1.33	51.51 ± 0.99	63.72 ± 5.34	64.32 ± 11.20	72.78 ± 8.53	80.62 ± 0.48
5/S/450-80	94.90 ± 3.86	55.23 ± 0.62	51.29 ± 5.95	63.29 ± 0.37	74.71 ± 1.84	78.20 ± 8.66
5/NS/450-90	97.77 ± 2.05	55.01 ± 2.01	62.14 ± 2.25	69.35 ± 1.75	70.93 ± 0.49	79.90 ± 4.95
5/S/450-90	96.75 ± 3.70	55.84 ± 0.95	59.58 ± 3.99	70.55 ± 7.53	74.11 ± 17.96	81.62 ± 0.99
5/NS/900-45	94.82 ± 3.15	54.52 ± 0.96	59.64 ± 4.76	65.47 ± 2.98	69.96 ± 3.36	75.70 ± 3.15
5/S/900-45	95.00 ± 6.76	54.13 ± 0.37	62.05 ± 3.44	71.01 ± 2.18	71.60 ± 7.06	71.74 ± 4.77
5/NS/900-50	100.00 ± 0	52.96 ± 0.15	60.61 ± 3.40	67.69 ± 12.64	67.90 ± 6.24	68.91 ± 25.63
5/S/900-50	87.76 ± 13.32	54.77 ± 6.40	60.68 ± 14.88	60.86 ± 8.25	72.55 ± 6.45	72.60 ± 4.51
Effect of BSG Content						
0%	67.65 ^a^	46.24 ^a^	49.10 ^a^	58.72 ^b^	70.15 ^c^	77.12 ^b^
1%	74.70 ^b^	49.93 ^b^	49.63 ^a^	55.03 ^a^	63.70 ^a^	77.17 ^b^
3%	90.67 ^c^	50.65 ^c^	56.12 ^b^	61.21 ^c^	64.81 ^b^	75.92 ^a^
5%	95.73 ^d^	54.88 ^d^	59.96 ^c^	66.57 ^d^	71.82 ^d^	76.16 ^a^
Significance (*p* < 0.05)	*	*	*	*	*	*
Effect of Sonication						
Not Sonicated	83.80 ^b^	49.35 ^a^	52.81 ^a^	59.51 ^a^	67.10 ^a^	77.70 ^b^
Sonicated	80.55 a	51.49 ^b^	54.59 ^b^	61.25 ^b^	68.15 ^b^	75.49 ^a^
Significance (*p* < 0.05)	*	*	*	*	*	*
Effect of Microwave Treatment						
450 W-80 s	84.65 ^c^	50.24 ^a^	53.38 ^a^	59.68 ^ab^	69.02 ^b^	76.16 ^b^
450 W-90 s	81.58 ^b^	51.09 ^b^	53.40 ^a^	59.88 ^b^	68.24 ^b^	78.52 ^c^
900 W-45 s	81.59 ^b^	50.10 ^a^	54.49 ^b^	59.34 ^a^	64.70 ^a^	75.39 ^a^
900 W-50 s	80.89 ^a^	50.28 ^a^	53.52 ^a^	62.63 ^c^	68.55 ^ab^	76.30 ^b^
Significance (*p* < 0.05)	*	*	*	*	*	*

In column, different letters correspond to statistically significant values. * indicate significant differences within groups of samples.

**Table 7:** Tensile properties of the thermoplastic starch–BSG composite films.

Type	Young’s Modulus (N/mm^2^)	Tensile Strength (N/mm^2^)	Strain at Maximum Force (%)
0/NS/450-80	4.19 ± 0.44	0.37 ± 0.04	13.34 ± 2.92
0/S/450-80	3.08 ± 0.39	0.34 ± 0.05	16.05 ± 2.59
0/NS/450-90	4.10 ± 0.68	0.37 ± 0.07	15.20 ± 3.51
0/S/450-90	3.82 ± 0.37	0.42 ± 0.08	17.23 ± 3.56
0/NS/900-45	4.88 ± 1.58	0.42 ± 0.11	14.67 ± 3.42
0/S/900-45	5.94 ± 1.28	0.51 ± 0.13	14.25 ± 2.58
0/NS/900-50	5.48 ± 0.71	0.51 ± 0.07	17.47 ± 3.31
0/S/900-50	7.60 ± 1.59	0.73 ± 0.13	17.80 ± 3.73
1/NS/450-80	6.52 ± 0.92	0.57 ± 0.15	15.23 ± 4.40
1/S/450-80	6.17 ± 0.81	0.51 ± 0.12	13.25 ± 2.13
1/NS/450-90	6.57 ± 0.66	0.53 ± 0.03	12.60 ± 1.92
1/S/450-90	5.83 ± 1.23	0.47 ± 0.09	12.03 ± 1.58
1/NS/900-45	6.44 ± 0.70	0.51 ± 0.06	12.37 ± 2.43
1/S/900-45	7.84 ± 0.69	0.62 ± 0.07	13.43 ± 2.64
1/NS/900-50	6.42 ± 0.79	0.51 ± 0.11	13.06 ± 2.95
1/S/900-50	8.63 ± 1.01	0.60 ± 0.09	12.30 ± 2.09
3/NS/450-80	3.39 ± 0.50	0.31 ± 0.06	14.05 ± 2.15
3/S/450-80	3.16 ± 0.41	0.25 ± 0.04	11.12 ± 1.97
3/NS/450-90	2.94 ± 0.38	0.26 ± 0.03	13.20 ± 1.81
3/S/450-90	3.21 ± 0.43	0.22 ± 0.06	9.70 ± 2.22
3/NS/900-45	4.03 ± 0.25	0.35 ± 0.04	14.02 ± 1.78
3/S/900-45	4.61 ± 0.52	0.39 ± 0.06	13.58 ± 1.58
3/NS/900-50	4.16 ± 0.33	0.35 ± 0.03	14.30 ± 1.56
3/S/900-50	4.55 ± 0.34	0.38 ± 0.04	13.72 ± 1.44
5/NS/450-80	2.97 ± 1.31	0.22 ± 0.10	11.35 ± 1.36
5/S/450-80	2.89 ± 0.92	0.21 ± 0.06	12.10 ± 1.79
5/NS/450-90	2.28 ± 0.42	0.18 ± 0.03	12.58 ± 1.31
5/S/450-90	2.31 ± 0.53	0.17 ± 0.02	11.38 ± 1.60
5/NS/900-45	3.63 ± 1.41	0.26 ± 0.08	12.50 ± 0.98
5/S/900-45	3.04 ± 0.23	0.21 ± 0.02	12.80 ± 2.23
5/NS/900-50	2.41 ± 0.31	0.18 ± 0.04	12.05 ± 2.08
5/S/900-50	3.68 ± 0.33	0.24 ± 0.04	11.20 ± 1.63
Effect of BSG Content			
0%	4.89 ^c^	0.46 ^c^	15.75 ^c^
1%	6.80 ^d^	0.54 ^d^	13.03 ^b^
3%	3.76 ^b^	0.31 ^b^	12.95 ^b^
5%	2.90 ^a^	0.21 ^a^	11.97 ^a^
Significance (*p* < 0.05)	*	*	*
Effect of Sonication			
Not Sonicated	4.40 ^a^	0.37 ^a^	13.62 ^b^
Sonicated	4.77 ^b^	0.39 ^b^	13.25 ^a^
Significance (*p* < 0.05)	*	*	*
Effect of Microwave Treatment			
450 W-80 s	4.05 ^b^	0.35 ^b^	13.30 ^ab^
450 W-90 s	3.88 ^a^	0.33 ^a^	12.99 ^a^
900 W-45 s	5.05 ^c^	0.41 ^c^	13.45 ^b^
900 W-50 s	5.37 ^d^	0.44 ^d^	13.99 ^c^
Significance (*p* < 0.05)	*	*	*

In column, different letters correspond to statistically significant values. * indicate significant differences within groups of samples.

**Table 8:** Equations and statistics of the quadratic models (*p* < 0.05) that describe the effects of BSG content (BSG), microwave treatments (MW), and possible sonication (US) on several characteristics of the thermoplastic starch–BSG composite films.

Equations								
Density = 0.42161 + 0.38937*BSG + 0.00001*MW − 1.10127*US − 0.02830*BSG^2^ − 0.00001*BSG*MW + 0.05068*BSG*US + 0.00002*MW*US								
UV_A blocking = 10.6283*BSG + 0.0065*MW + 12.3882*US − 1.5440*BSG^2^ − 0.00000007*MW2 − 0.0003*MW*US								
UV_B blocking = −188.86 + 21.686*BSG + 0.012*MW + 19.613*US − 2.932*BSG^2^ − 0.0000001*MW^2^ − 0.0004*MW*US								
Water absorption = 93.67707 + 5.97604*BSG − 0.00062*MW − 0.88280*BSG^2^ + 0.00011*BSG*MW − 0.00008*MW*US								
Water solubility = 46.13619 + 1.42326*BSG + 0.00005*MW*US								
Weight loss at 3 days = 46.07054 + 2.98232*BSG − 1.29109*BSG*US + 0.00012*MW*US								
Weight loss at 7 days = 192.2305 + 4.1537*BSG − 0.0078*MW + 25.4993*US + 0.5781*BSG^2^ + 0.0000001*MW^2^ − 0.0001*BSG*MW + −0.6266*BSG*US − 0.0006*MW*US								
Weight loss at 10 days = 249.4446 − 0.0091*MW + 1.2051*BSG^2^ + 0.0000001*MW^2^ − 0.0001*BSG*MW + 0.3759*BSG*US								
Weight loss at 14 days = 56.69738 + 10.12015*BSG + 0.00055*MW − 3.94275*US − 0.00027*BSG*MW + 0.77960*BSG*US								
Young’s modulus = 34.6128 + 2.57098*BSG − 0.00167*MW − 7.07627*US − 0.13288*BSG^2^ + 0.00000002*MW^2^ − 0.00006*BSG*MW + 0.00018*MW*US								
Tensile strength = 3.187055 + 0.181482*BSG − 0.000151*MW − 0.486413*US − 0.00791*BSG^2^ + 0.000000002*MW^2^ − 0.000005*BSG*MW − 0.015258*BSG*US + 0.000013*MW*US								
Strain at maximum force = 56.09974 − 0.00219*MW + 0.17428*BSG^2^ + 0.00000002*MW^2^ − 0.00003*BSG*MW − 0.21204*BSG*US								
Statistics of the quadratic models								
Dependent Variable	R^2^	Model			Residual			F
		SS	df	MS	SS	df	MS	
Density (g/cm^3^)	0.499	11.449	7	1.636	11.513	472	24.392	67.053
UV_A blocking (%)	0.757	22,609.37	6	3768.228	7292.297	473	15.417	244.418
UV_B blocking (%)	0.856	10,8945.80	7	15,563.680	18,468.66	472	39.128	397.758
Water absorption (%)	0.893	64,699.17	5	12,939.830	7739.574	474	16.328	792.483
Water solubility (%)	0.567	4154.04	2	2077.021	3168.500	477	6.643	312.649
Weight loss 3 days (%)	0.532	10,844.30	3	3614.768	9543.211	476	20.049	180.299
Weight loss 7 days (%)	0.527	9418.35	8	1177.293	8441.158	471	17.922	65.691
Weight loss 10 days (%)	0.360	6410.72	5	1282.145	11,398.010	474	24.064	53.319
Weight loss 14 days (%)	0.187	2286.13	5	457.226	9959.659	474	21.012	21.760
Young’s modulus (N/mm^2^)	0.625	945.67	7	135.094	568.424	472	1.204	112.178
Tensile strength (N/mm^2^)	0.736	8.27	8	1.034	2.975	471	0.006	163.710
Strain at maximum force (%)	0.327	885.55	5	177.109	1818.637	474	3.837	146.161

SS: sum of squares; df: degree of freedom; MS: mean square.

## Data Availability

Data will be made available on request.

## Supplementary Materials

The following supporting information can be downloaded at https://www.mdpi.com/article/10.3390/polym18080967/s1. Figure S1: Iso-response surfaces.

## Abbreviations

The following abbreviations are used in this manuscript:

BSG	Brewers’ spent grain
L*	Lightness
a*	Position of color between red and green
b*	Position of color between yellow and blue
BI	Browning index
ΔE	Total color difference between the sample and a standard
SEM	Scanning electron microscopy
ANOVA	Analysis of variance
PCA	Principal component analysis

## References

[1] Guillard V., Gaucel S., Fornaciari C., Angellier-Coussy H., Buche P., Gontard N. (2020). The next generation of sustainable food packaging to preserve our environment in a circular economy context. Front. Nutr..

[2] Regulation (EU) 2025/40 on “Packaging and Packaging Waste”, Amending Regulation (EU) 2019/1020 and Directive (EU) 2019/904, and Repealing Directive 94/62/EC, Official Journal L, 2025/40, 22.1.2025. http://data.europa.eu/eli/reg/2025/40/oj.

[3] Geyer R., Jambeck J.R., Law K.L. (2017). Production, use, and fate of all plastics ever made. Sci. Adv..

[4] ING Economics Department (2019). Plastic Packaging in the Food Sector—Six Ways to Tackle the Plastic Puzzle. https://think.ing.com/uploads/reports/ING_-_The_plastic_puzzle_-_December_2019_%28003%29.pdf.

[5] FAO (2017). The Future of Food and Agriculture—Trends and Challenges.

[6] Chauhan C., Dhir A., Akram M.U., Salo J. (2021). Food loss and waste in food supply Chains. A systematic literature review and framework development approach. J. Clean. Prod..

[7] Versino F., Ortega F., Monroy Y., Rivero S., López O.V., García M.A. (2023). Sustainable and bio-based food packaging: A review on past and current design innovations. Foods.

[8] Babbitt C.W., Neff R.A., Roe B.E., Siddiqui S., Chavis C., Trabold T.A. (2022). Transforming wasted food will require systemic and sustainable infrastructure innovations. Curr. Opin. Environ. Sustain..

[9] Qazanfarzadeh Z., Ganesan A.R., Mariniello L., Conterno L., Kumaravel V. (2023). Valorization of brewer’s spent grain for sustainable food packaging. J. Clean. Prod..

[10] European Commission’s Directorate-General Environment (2011). Plastic waste: Ecological and human health impacts. Sci. Environ. Pol..

[11] Pira S. (2013). The Future of Bioplastics for Packaging to 2020: Global Market Forecasts. Smithers Pira.

[12] Morro A., Catalina F., Corrales T., Pablos J.L., Marin I., Abrusci C. (2016). New blends of ethylene-butyl acrylate copolymers with thermoplastic starch. Characterization and bacterial biodegradation. Carb. Polym..

[13] Kumar Y., Shikha D., Guzmán-Ortiz F.A., Sharanagat V.S., Kumar K., Saxena D.C., Sharanagat V.S., Saxena D.C., Kumar K., Kumar Y. (2023). Starch: Current Production and Consumption Trends. Starch: Advances in Modifications, Technologies and Applications.

[14] Hejna A. (2022). More than just a beer—The potential applications of by-products from beer manufacturing in polymer technology. Emerg. Mat..

[15] Assandri D., Pampuro N., Zara G., Cavallo E., Budroni M. (2021). Suitability of composting process for the disposal and valorization of brewer’s spent grain. Agriculture.

[16] Kavalopoulos M., Stoumpou V., Christofi A., Mai S., Barampouti E.M., Moustakas K., Malamis D., Loizidou M. (2021). Sustainable valorisation pathways mitigating environmental pollution from brewers’ spent grains. Environ. Poll..

[17] Zeko-Pivač A., Tišma M., Žnidaršič-Plazl P., Kulisic B., Sakellaris G., Hao J., Planinić M. (2022). The potential of brewer’s spent grain in the circular bioeconomy: State of the art and future perspectives. Front. Bioeng. Biotechnol..

[18] Amoriello T., Ciccoritti R. (2021). Sustainability: Recovery and reuse of brewing-derived by-products. Sustainability.

[19] Jaguey-Hernández Y., Tapia-Ignacio C., Aguilar-Arteaga K., González-Olivares L.G., Castañeda-Ovando E.P., Cruz-Cansino N., Ojeda-Ramirez D., Castañeda-Ovando A. (2023). Thermoplastic biofilms obtained from an arabinoxylan-rich fraction from brewers’ spent grain: Physicochemical characterization and thermal analysis. Biomass Conv. Biorefin..

[20] Liu L., Chen M., Coldea T.E., Yang H., Zhao H. (2023). Emulsifying properties of arabinoxylans derived from brewers’ spent grain by ultrasound-assisted extraction: Structural and functional properties correlation. Cellulose.

[21] Proaño J.L., Salgado P.R., Cian R.E., Mauri A.N., Drago S.R. (2020). Physical, structural and antioxidant properties of brewer’s spent grain protein films. J. Sci. Food Agric..

[22] Castanho M.N., de Souza do Prado K., Faulstich de Paiva J.M. (2022). Developing thermoplastic corn starch composites filled with brewer’s spent grain for applications in biodegradable films. Polym. Comp..

[23] Shroti G.K., Saini C.S. (2022). Development of edible films from protein of brewer’s spent grain: Effect of pH and protein concentration on physical, mechanical and barrier properties of films. Appl. Food Res..

[24] Taner O.O., Ekici L., Akyuz L. (2023). CMC-based edible coating composite films from brewer’s spent grain waste: A novel approach for the fresh strawberry package. Polym. Bull..

[25] Mendes J.F., Norcino L.B., Martins H.H., Manrich A., Otoni C.G., Carvalho E.E.N., Piccolli R.H., Oliveira J.E., Pinheiro A.C.M., Mattoso L.H.C. (2021). Development of quaternary nanocomposites made up of cassava starch, cocoa butter, lemongrass essential oil nanoemulsion, and brewery spent grain fibers. J. Food Sci..

[26] Hejna A., Barczewski M., Kosmela P., Mysiukiewicz O., Sulima P., Przyborowski J.A., Kowalkowska-Zedler D. (2022). Mater-Bi/brewers’ spent grain biocomposites—Novel approach to plant-based waste filler treatment by highly efficient thermomechanical and chemical methods. Materials.

[27] Soykeabkaew N., Thanomsilp C., Suwantong O. (2015). A review: Starch-based composite foams. Compos. Part A Appl. Sci. Manuf..

[28] Quinez-Molina A.I., Oliveira-Salmazo L., Lopez-Gil A., Rodríguez-Pérez M.A. (2024). Novel flexible and active expanded-starch films enriched with Agrifood waste via microwave irradiation. Future Foods.

[29] Li W., Yang H., Coldea T.E., Zhao H. (2021). Modification of structural and functional characteristics of brewer’s spent grain protein by ultrasound assisted extraction. LWT—Food Sci. Technol..

[30] Palav T., Seetharaman K. (2006). Mechanism of starch gelatinization and polymer leaching during microwave heating. Carbohyd. Polym..

[31] Müller C.M.O., Yamashita F., Laurindo J.B. (2008). Evaluation of the effects of glycerol and sorbitol concentration and water activity on the water barrier properties of cassava starch films through a solubility approach. Carbohyd. Polym..

[32] Garuti G.L., Freitas R.R.M., Lima V.H., Carmo K.P., Pádua F.A., Botaro V.R. (2024). Nanocellulose reinforced starch biocomposite films via tape-casting technique. Polím. Ciênc. Tecnol..

[33] Guzman-Puyol S., Hierrezuelo J., Benítez J.J., Tedeschi G., Porras-Vázquez J.M., Heredia A., Athanassiou A., Romero D., Heredia-Guerrero J.A. (2022). Transparent, UV-blocking, and high barrier cellulose-based bioplastics with naringin as active food packaging materials. Int. J. Biol. Macromol..

[34] Zhao J., Wang Y., Liu C. (2022). Film transparency and opacity measurements. Food Anal. Meth..

[35] Tanetrungroj Y., Prachayawarakorn J. (2018). Effect of dual modification on properties of biodegradable crosslinked-oxidized starch and oxidized-crosslinked starch films. Int. J. Biol. Macromol..

[36] Lopes P.F.N., de Medeiros Felipe A.T., de Medeiros F.G.M., Rocha Bastos do Socorro M., Albuquerque Mattos A.L., Matsui K.N., Hoskin R.T. (2025). Biodegradable cassava starch-based films formulated with coconut oil for sustainable food packaging. Food Sci. Eng..

[37] (2012). Standard Test Method for Tensile Properties of Thin Plastic Sheeting.

[38] Sarmiento-Gaviria M.F., Escobar-Mora N., Hoyos-Palacio L.M., Espinel-Blanco E.E. (2019). Synthesis and mechanical characterization of a non-woven nanofiber by the electrospinning technique. Dyna.

[39] Amini A.M., Razavi S.M.A., Mortazavi S.A. (2015). Morphological, physicochemical, and viscoelastic properties of sonicated corn starch. Carbohyd. Polym..

[40] Nochi Castro L.E., Saragiotto Colpini L.M. (2021). All-around characterization of brewer’ spent grain. Eur. Food Res. Technol..

[41] Edhirej A., Sapuan S.M., Jawaid M., Zahari N. (2017). Preparation and characterization of cassava bagasse reinforced thermoplastic cassava starch. Fibers Polym..

[42] Devi L.M., Lalnunthari C., Badwaik L.S. (2019). Direct transformation of muskmelon seeds meal into biodegradable films and their characterization. J. Polym. Environ..

[43] Mazela B., Perdoch W., Peplińska B., Zieliński M. (2020). Influence of chemical pre-treatments and ultrasonication on the dimensions and appearance of cellulose fibers. Materials.

[44] Hejna A., Cieśliński H., Skórczewska K., Kosmela P., Aniśko-Michalak J., Piasecki A., Barczewski M. (2025). The impact of brewers’ spent grain type on the structure and performance of poly(ε-caprolactone)-based composites. Cellulose.

[45] Anderson C., Simsek S. (2018). What are the characteristics of arabinoxylan gels?. Food Nutr. Sci..

[46] Sanyang M.L., Sapuan S.M., Jawaid M., Ishak M.R., Sahari J. (2016). Effect of plasticizer type and concentration on physical properties of biodegradable films based on sugar palm (*Arenga pinnata*) starch for food packaging. J Food Sci. Technol..

[47] Patrignani M., Brantsen J.F., Awika J.M., Conforti P.A. (2021). Application of a novel microwave energy treatment on brewers’ spent grain (BSG): Effect on its functionality and chemical characteristics. Food Chem..

[48] Fărcaș A.C., Socaci S.A., Chiș M.S., Pop O.L., Fogarasi M., Păucean A., Igual M., Michiu D. (2021). Reintegration of brewers spent grains in the food chain: Nutritional, functional and sensorial aspects. Plants.

[49] Liu J., Zhang J., Wang W., Hou H. (2021). Effects of microwave treatment on the stability and antioxidant capacity of a functional wheat bran. Food Sci. Nutr..

[50] Liu H., Xie F., Yu L., Chen L., Li L. (2009). Thermal processing of starch-based polymers. Prog. Polym. Sci..

[51] Kai D., Tan M.J., Chee P.L., Chua Y.K., Yap Y.L., Loh X.J. (2020). Towards lignin-based functional materials in a sustainable world. Polymers.

[52] Ludka F.R., Klosowski A.B., Almeida Camargo G., Silva Justo A., Assis Andrade E., Beltrame F.L., Bonametti Olivato J. (2024). Brewers’ spent grain extract as antioxidants in starch-based active biopolymers. Int. J. Food Sci. Technol..

[53] Shaw L.E., Lee D. (2009). Sonication of pulp and paper effluent. Ultrason. Sonochem..

[54] Fang S., Qiu W., Mei J., Xie J. (2020). Effect of sonication on the properties of flaxseed gum films incorporated with carvacrol. Int. J. Mol. Sci..

[55] McKenzie T.G., Karimi F., Ashokkumar M., Qiao G.G. (2019). Ultrasound and sonochemistry for radical polymerization: Sound synthesis. Chemistry.

[56] Lv S., Zhang Y., Gu J., Tan H. (2017). Biodegradation behavior and modelling of soil burial effect on degradation rate of PLA blended with starch and wood flour. Colloids Surf. B.

[57] Göpferich A. (1996). Mechanisms of polymer degradation and erosion. Biomaterials.

[58] Wang H., Wei D., Zheng A., Xiao H. (2015). Soil burial biodegradation of antimicrobial biodegradable PBAT films. Polym. Degrad. Stabil..

[59] Scarascia-Mugnozza G., Schettini E., Vox G., Malinconico M., Immirzi B., Pagliara S. (2006). Mechanical properties decay and morphological behaviour of biodegradable films for agricultural mulching in real scale experiment. Polym. Degrad. Stabil..

[60] Ma X., Yu J., Kennedy J.F. (2005). Studies on the properties of natural fibers-reinforced thermoplastic starch composites. Carbohydr. Polym..

[61] Azeredo H.M.C., Rosa M.F., Mattoso L.H.C. (2017). Nanocellulose in bio-based food packaging applications. Ind. Crops Prod..

[62] Asrofi M., Abral H., Kurnia Putra Y., Sapuan S.M., Kim H.-J. (2018). Effect of duration of sonication during gelatinization on properties of tapioca starch water hyacinth fiber biocomposite. Int. J. Biol. Macromol..

[63] Uygun E., Yildiz E., Sumnu G., Sahin S. (2020). Microwave pretreatment for the improvement of physicochemical properties of carob flour and rice starch–based electrospun nanofilms. Food Bioproc. Technol..

[64] Jia W., Gong R.H., Soutis C., Hogg P.J. (2014). Biodegradable fibre reinforced composites composed of polylactic acid and polybutylene succinate. Plast. Rub. Compos..

[65] Abral H., Putra G.J., Asrofi M., Park J.-W., Kim H.-J. (2018). Effect of vibration duration of high ultrasound applied to bio-composite while gelatinized on its properties. Ultrason. Sonochem..

