# Supplementary figures and images for: Ultrasound and Microwave Treatments to Produce Flexible Thermoplastic Starch–Brewers’ Spent Grain Composite Films
Source: Polymers (Basel). 2026 Apr 16;18(8):967. doi: 10.3390/polym18080967 (PMC13119927; doi:10.3390/polym18080967)

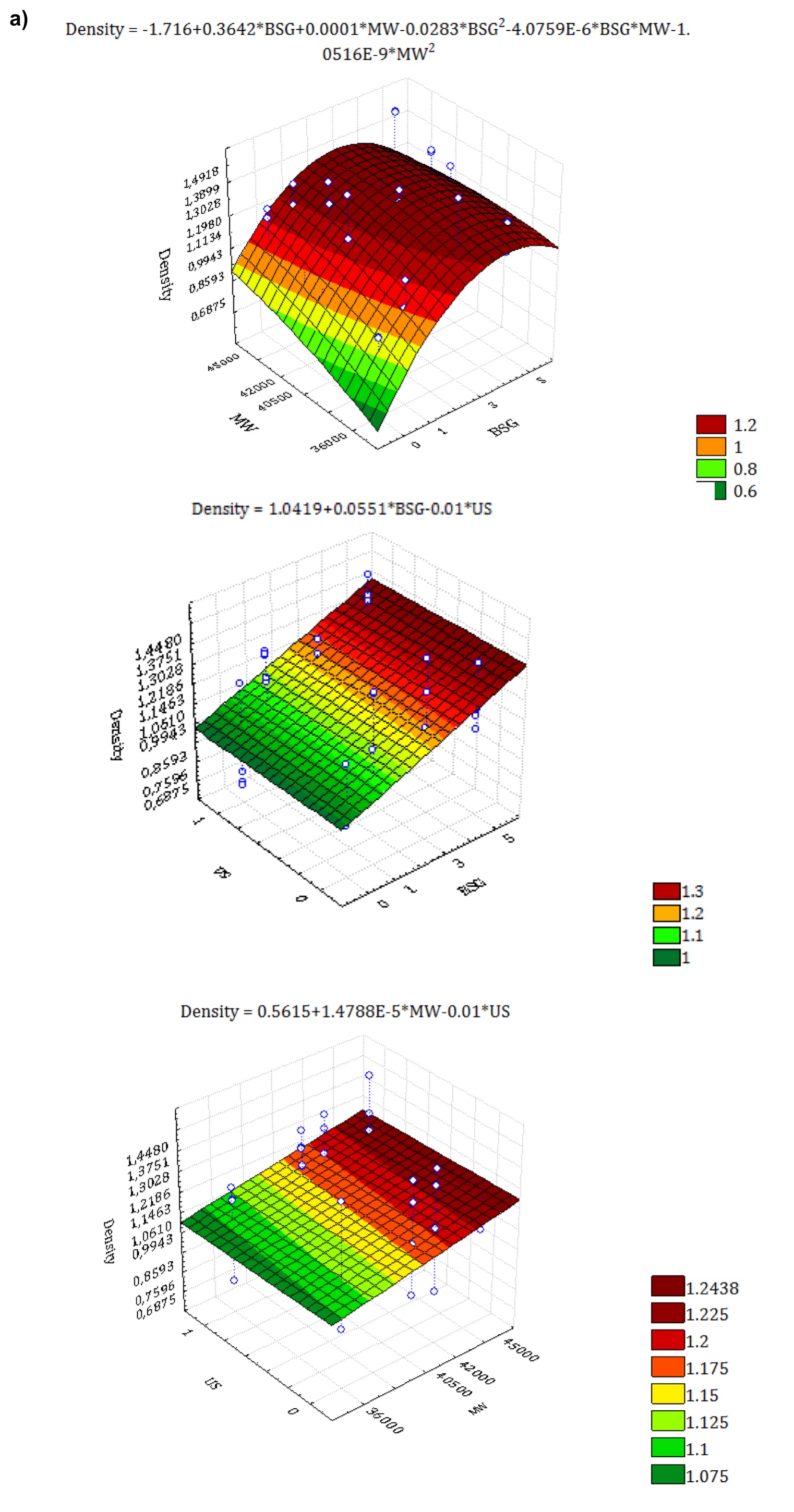

Supplement: Supplementary file 1 [file polymers-18-00967-s001.zip › Figure S1a.jpg]

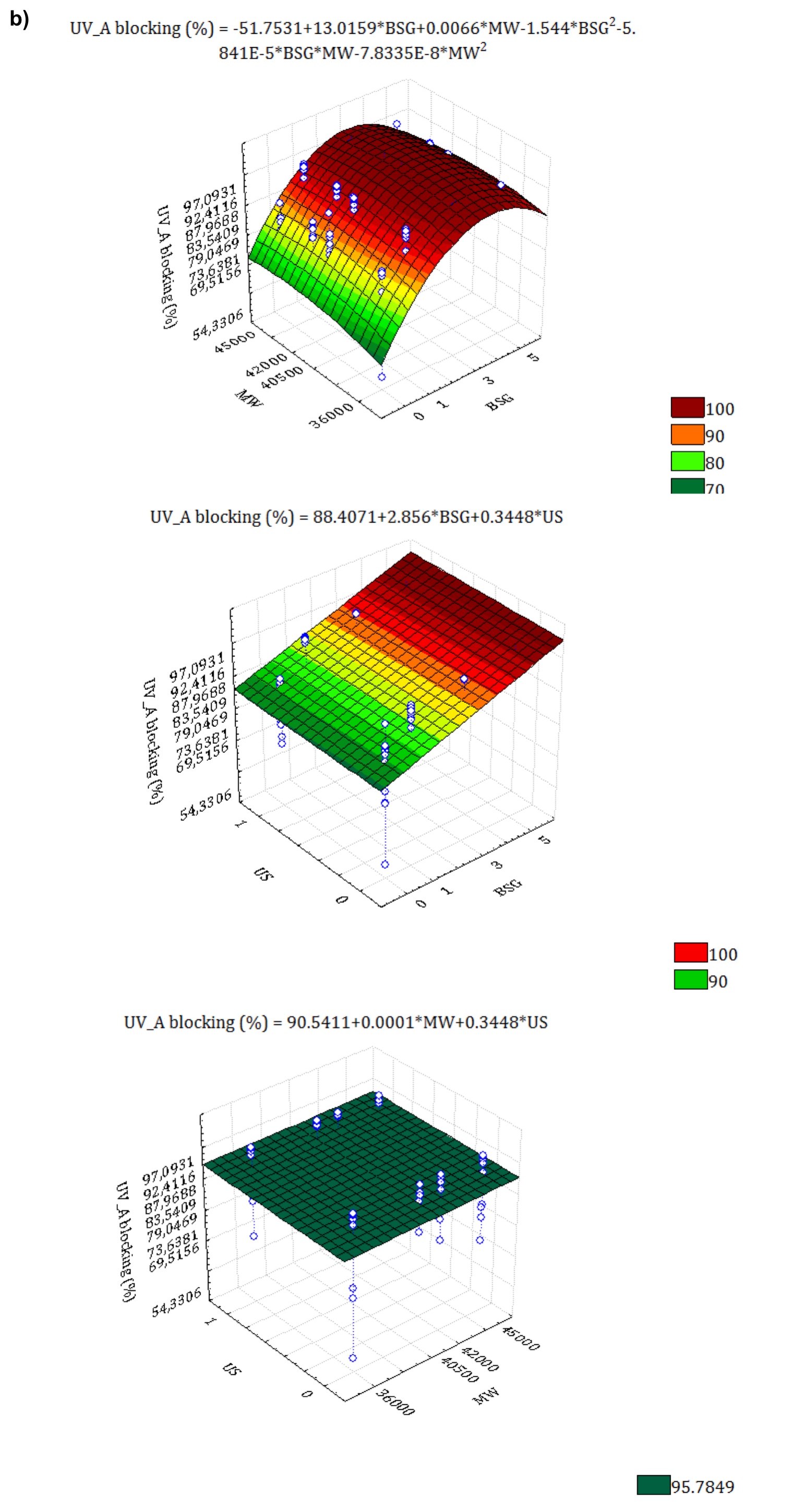

Supplement: Supplementary file 1 [file polymers-18-00967-s001.zip › Figure S1b.jpg]

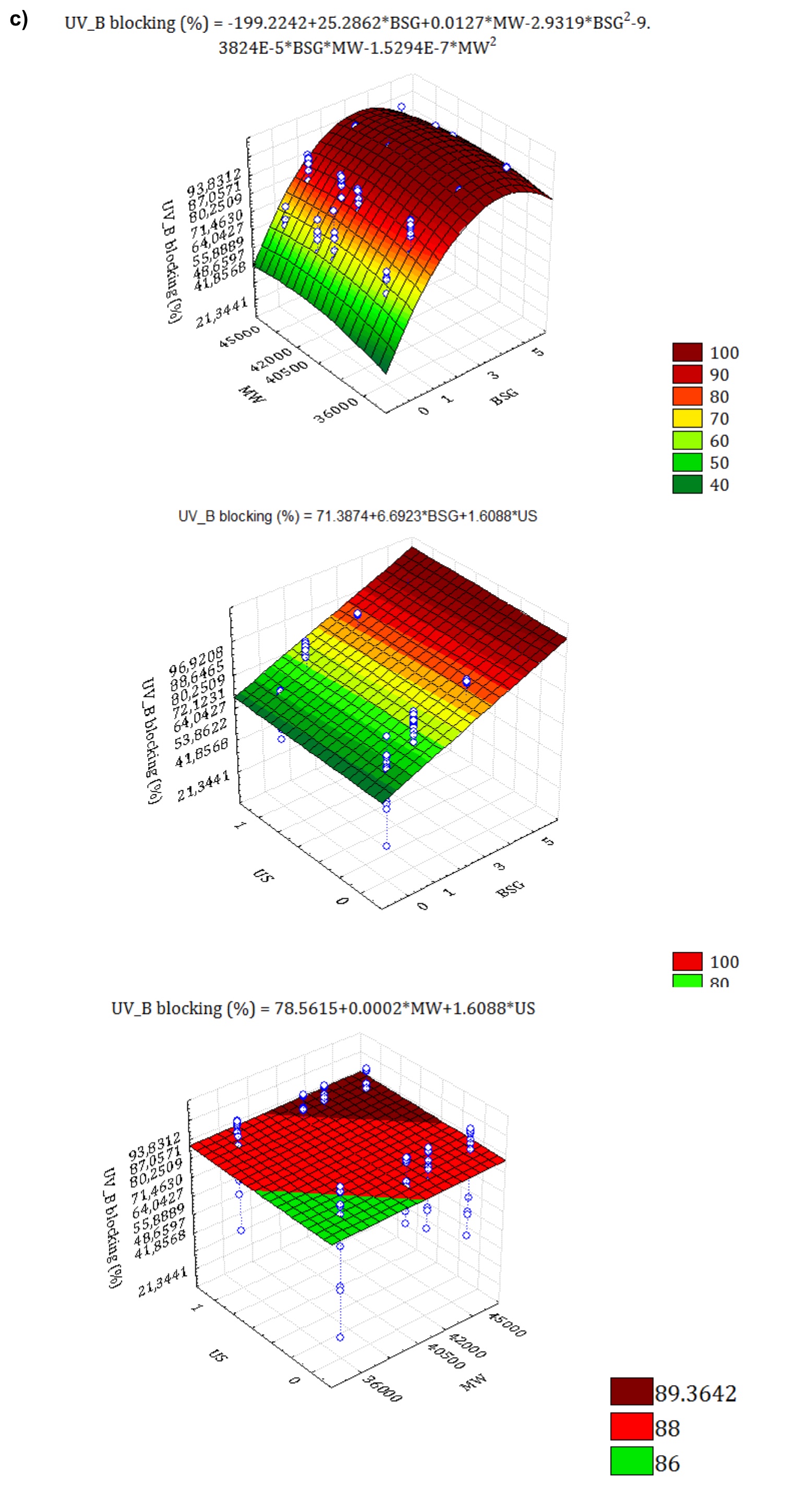

Supplement: Supplementary file 1 [file polymers-18-00967-s001.zip › Figure S1c.jpg]

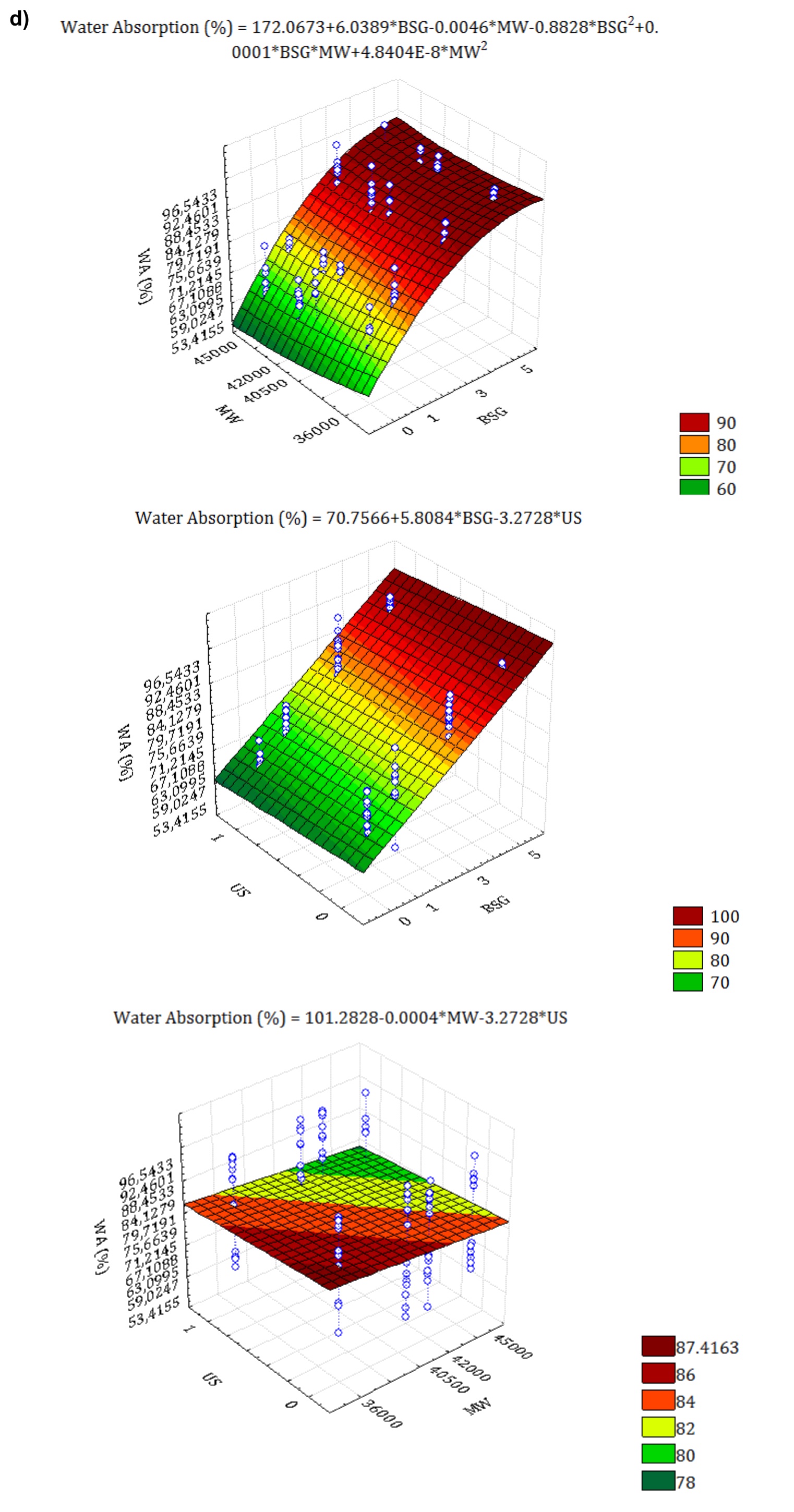

Supplement: Supplementary file 1 [file polymers-18-00967-s001.zip › Figure S1d.jpg]

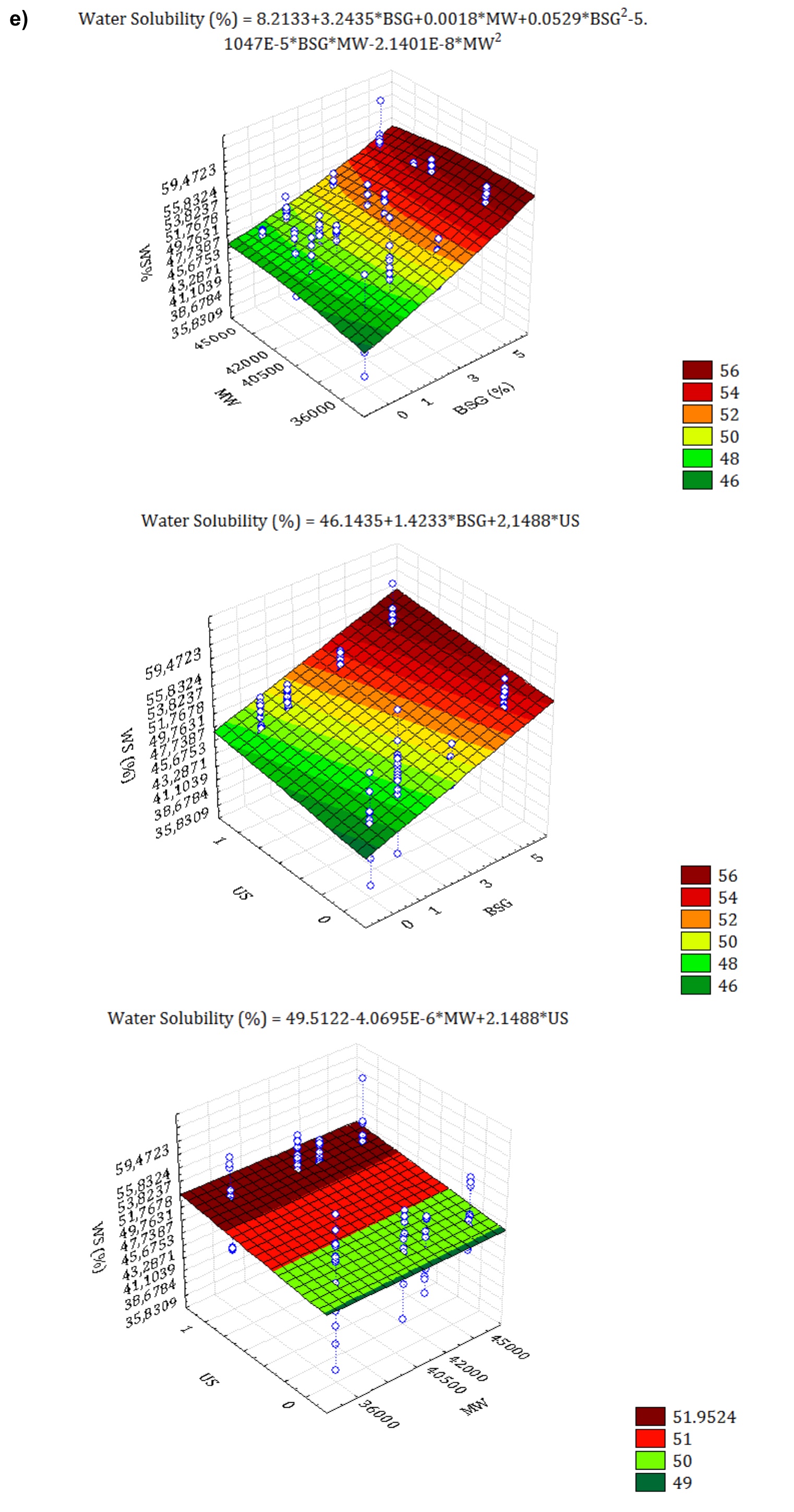

Supplement: Supplementary file 1 [file polymers-18-00967-s001.zip › Figure S1e.jpg]

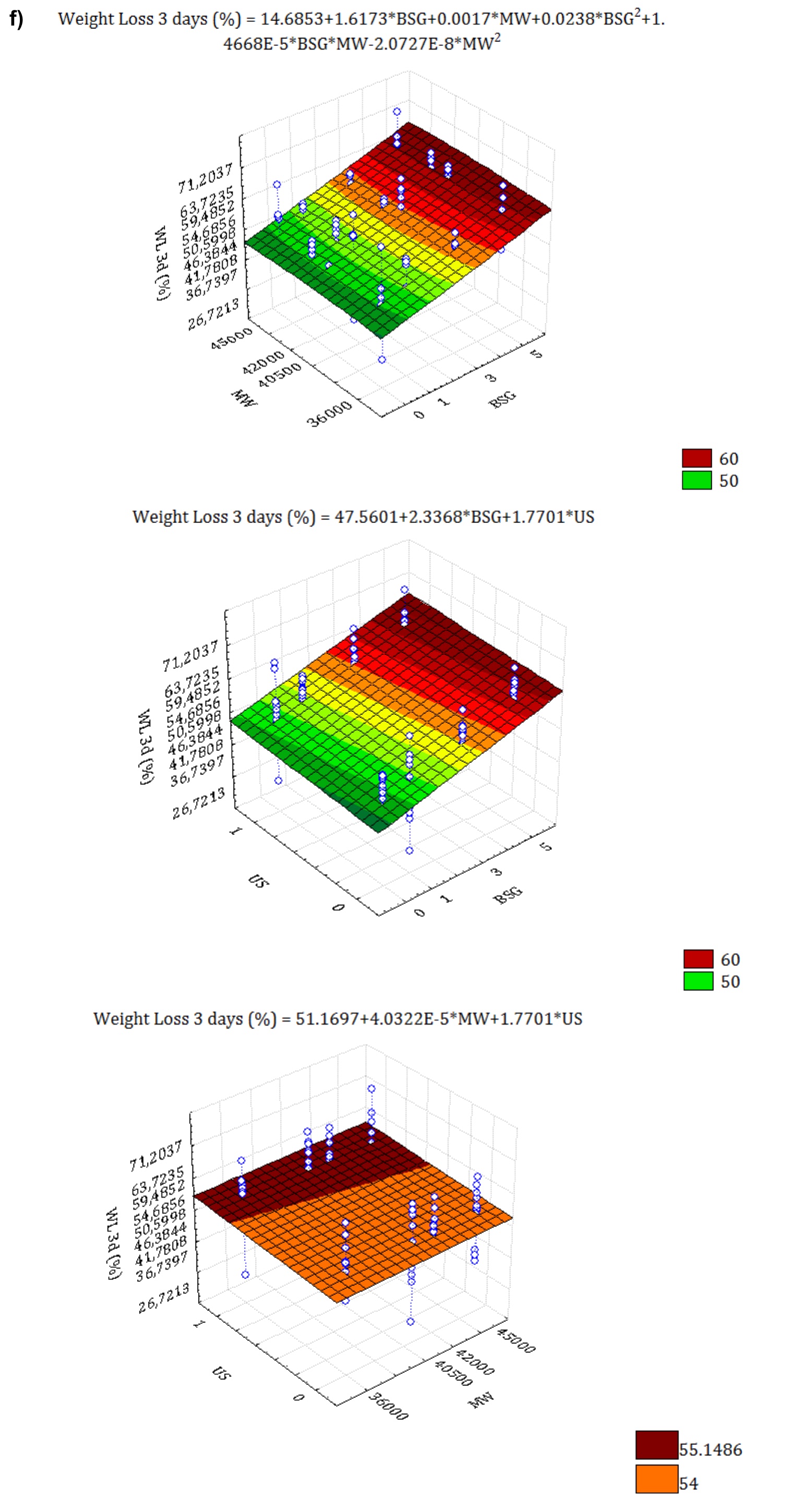

Supplement: Supplementary file 1 [file polymers-18-00967-s001.zip › Figure S1f.jpg]

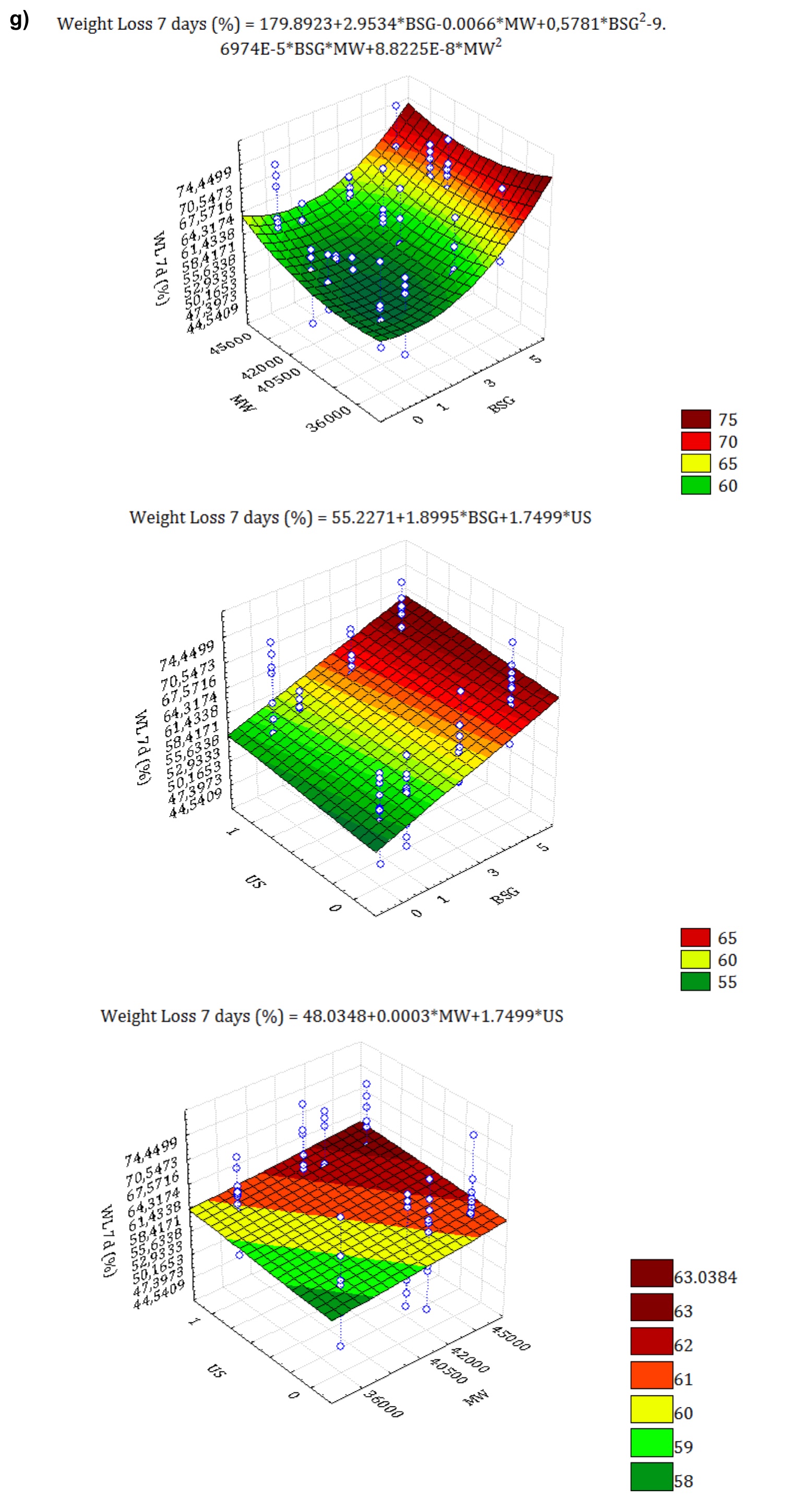

Supplement: Supplementary file 1 [file polymers-18-00967-s001.zip › Figure S1g.jpg]

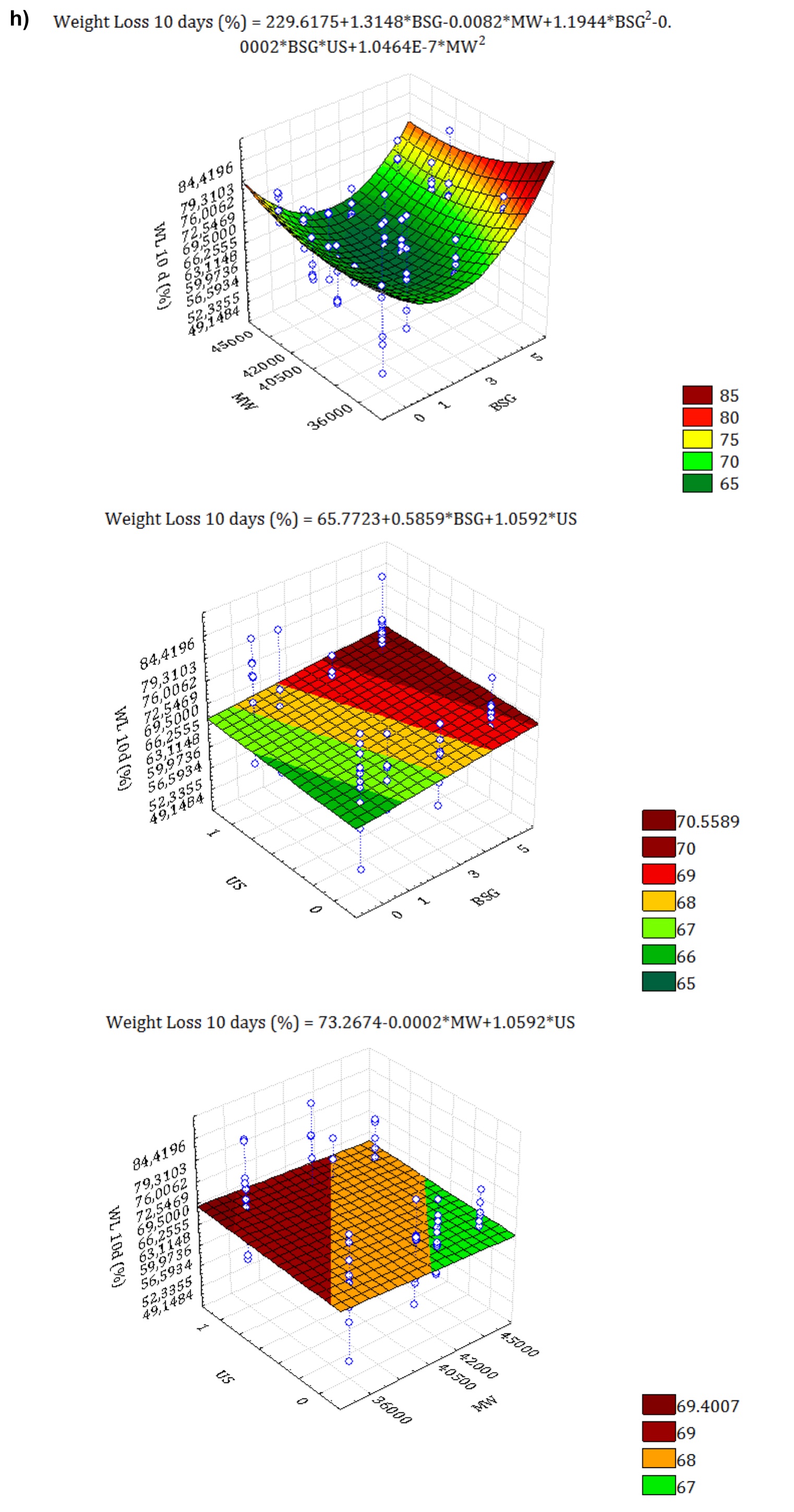

Supplement: Supplementary file 1 [file polymers-18-00967-s001.zip › Figure S1h.jpg]

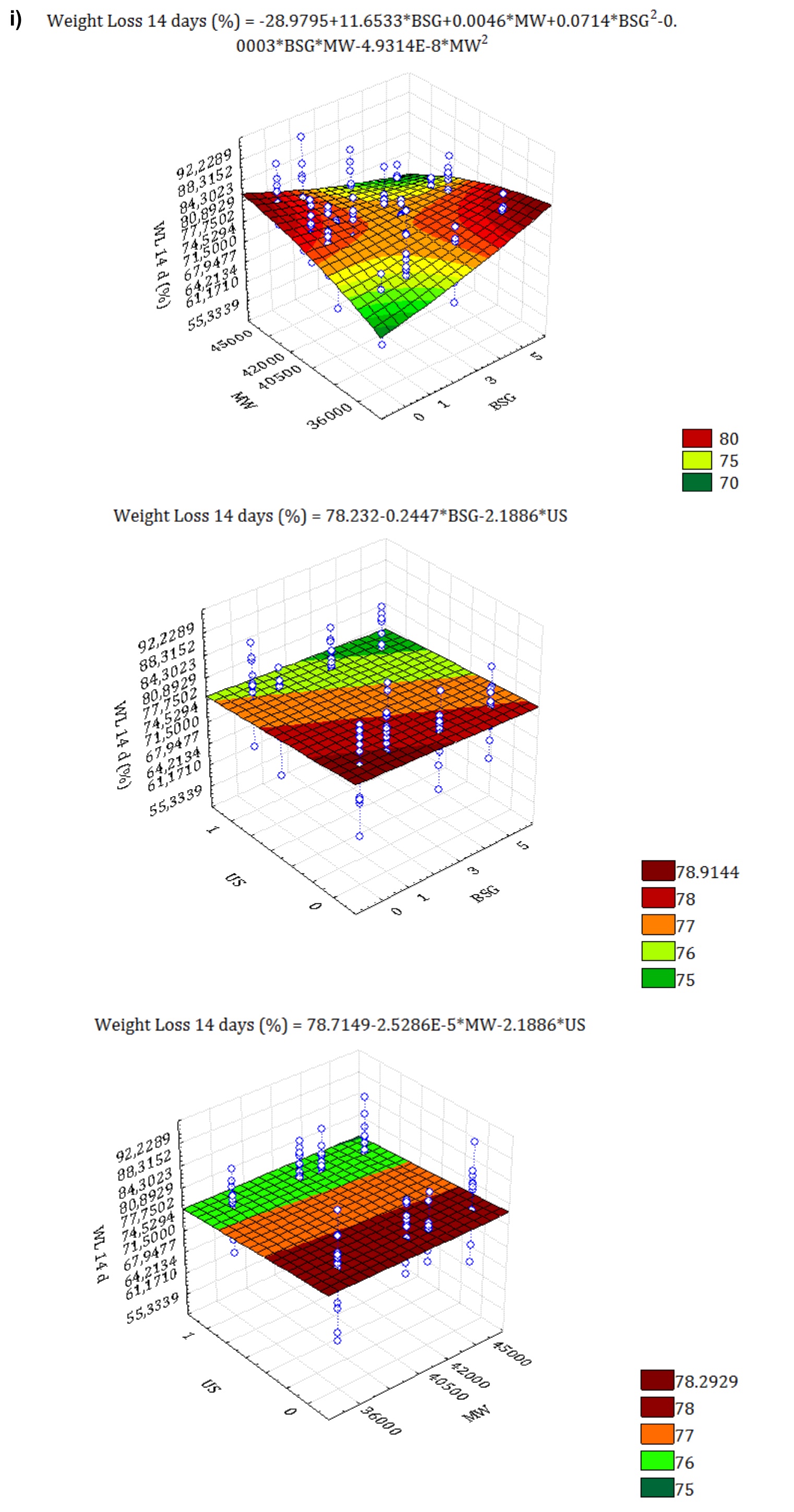

Supplement: Supplementary file 1 [file polymers-18-00967-s001.zip › Figure S1i.jpg]

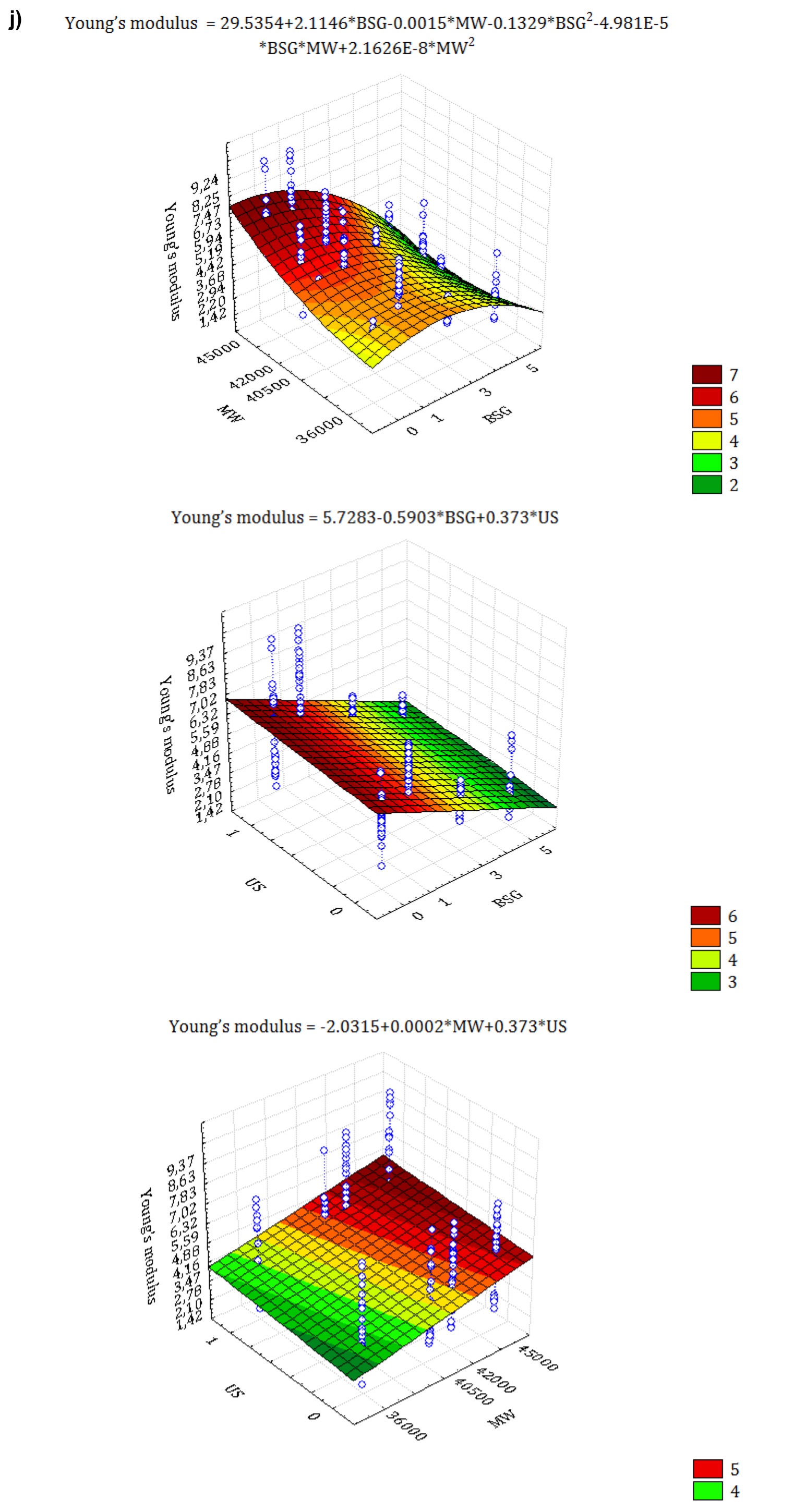

Supplement: Supplementary file 1 [file polymers-18-00967-s001.zip › Figure S1j.jpg]

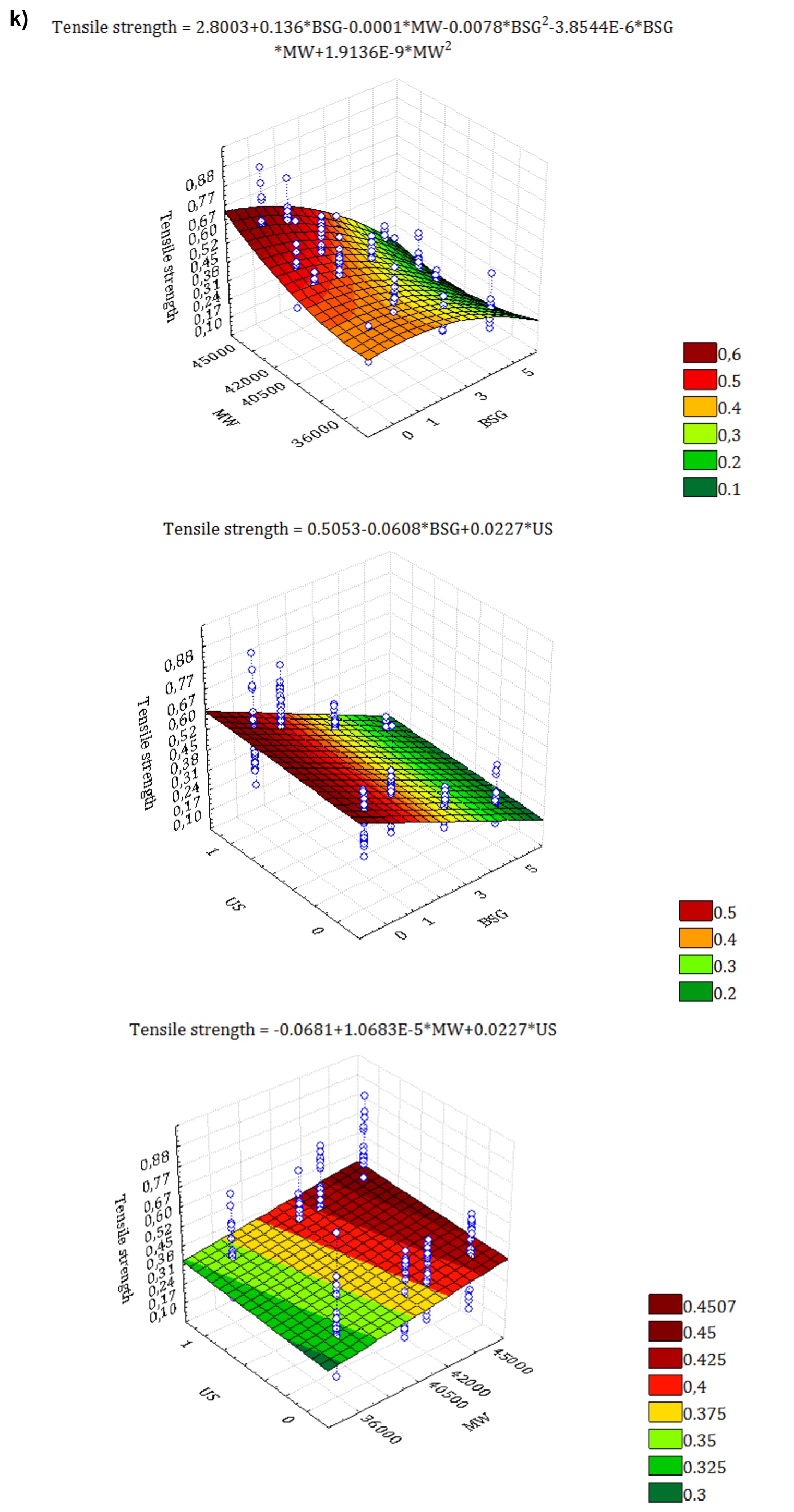

Supplement: Supplementary file 1 [file polymers-18-00967-s001.zip › Figure S1k.jpg]

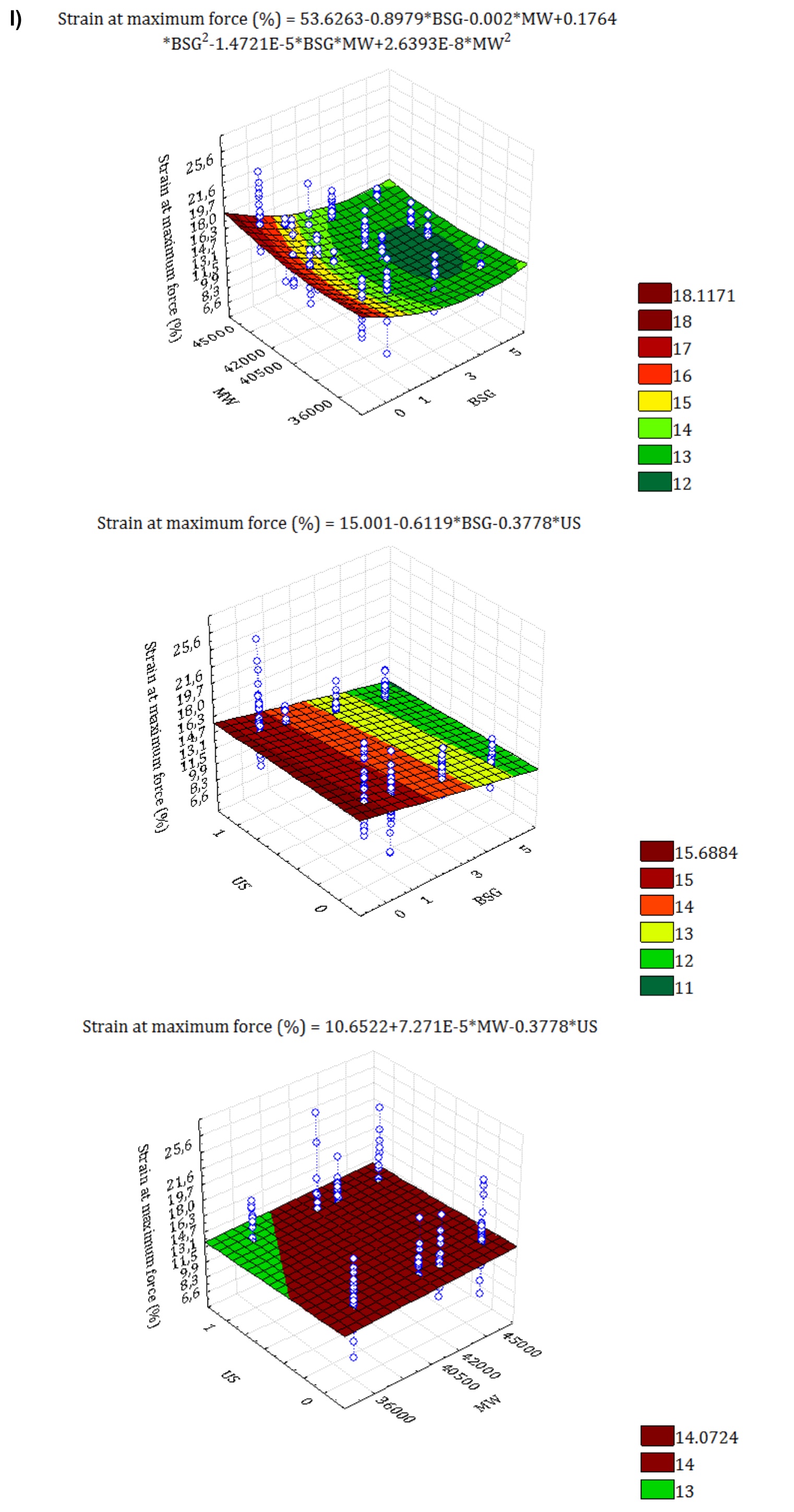

Supplement: Supplementary file 1 [file polymers-18-00967-s001.zip › Figure S1l.jpg]
